# Correction: Association of physical activity intensity and bout length with mortality: An observational study of 79,503 UK Biobank participants

**DOI:** 10.1371/journal.pmed.1004020

**Published:** 2022-06-01

**Authors:** Louise A. C. Millard, Kate Tilling, Tom R. Gaunt, David Carslake, Deborah A. Lawlor

The results of sensitivity analyses using the isometric log transformed activity variables were incorrect. The results have been corrected in S10–S12 Figs.

In the fifth paragraph of the Results section, the third sentence is incorrect. The paragraph should read:

Results of sensitivity analyses using “other day” imputed data were broadly consistent with the results of our main analyses using the complete days data (S4, S6, S7, and S9 Figs, S4–S6 Tables). Results of sensitivity analyses excluding BMI and ill health as covariates were comparable to our main analyses (S4–S6 Tables). Results using isometric log ratio transformed activity variables were largely consistent with our main analyses (S10–S12 Figs). However, unlike in our main analyses, we found some evidence that transferring time to very short MVPA bouts (1-9min duration) from longer MVPA bouts associates with lower mortality when larger quantities of time (e.g. 25mins) are transferred.

## Supporting information

S10 Fig Results of sensitivity analysis using isometric log ratio transformed activity variables: Association of time spent in activity categories, with all-cause mortality. (PDF)

S11 Fig Results of sensitivity analysis using isometric log ratio transformed activity variables: Association of time spent in MVPA bouts of a given length, with all-cause mortality. (PDF)

S12 Fig Results of sensitivity analysis using isometric log ratio transformed activity variables: Association of time spent in sedentary bouts of a given length, with all-cause mortality. (PDF)

## References

[1] Millard LAC, Tilling K, Gaunt TR, Carslake D, Lawlor DA (2021) Association of physical activity intensity and bout length with mortality: An observational study of 79,503 UK Biobank participants. PLoS Med 18(9): e1003757. 10.1371/journal.pmed.1003757 34525088 PMC8480840

