## [Editor Report · Decision Letter 0]

27 May 2020

Dear Dr Millard, 

Thank you for submitting your manuscript entitled "Association of changing physical activity intensity and bout length with mortality: a study of 79,507 participants in UK Biobank" for consideration by PLOS Medicine.

Your manuscript has now been evaluated by the PLOS Medicine editorial staff [as well as by an academic editor with relevant expertise] and I am writing to let you know that we would like to send your submission out for external peer review.

Kind regards,

Adya Misra, PhD,

Senior Editor

PLOS Medicine

---

## [Decision Letter · Decision Letter 1]

20 Aug 2020

Dear Dr. Millard,

Thank you very much for submitting your manuscript "Association of changing physical activity intensity and bout length with mortality: a study of 79,507 participants in UK Biobank" (PMEDICINE-D-20-02260R1) for consideration at PLOS Medicine. 

[LINK]

In light of these reviews, I am afraid that we will not be able to accept the manuscript for publication in the journal in its current form, but we would like to consider a revised version that addresses the reviewers' and editors' comments. Obviously we cannot make any decision about publication until we have seen the revised manuscript and your response, and we plan to seek re-review by one or more of the reviewers. 

We expect to receive your revised manuscript by Sep 10 2020 11:59PM. Please email us (plosmedicine@plos.org) if you have any questions or concerns.

We look forward to receiving your revised manuscript. 

Sincerely,

Adya Misra, PhD

Senior Editor 

PLOS Medicine

plosmedicine.org

Title 

Please revise your title according to PLOS Medicine's style. Your title must be nondeclarative and not a question. It should begin with main concept if possible. "Effect of" should be used only if causality can be inferred, i.e., for an RCT. Please place the study design ("A randomized controlled trial," "A retrospective study," "A modelling study," etc.) in the subtitle (ie, after a colon).

Abstract

* Please ensure that all numbers presented in the abstract are present and identical to numbers presented in the main manuscript text. * Please include the study design, population and setting, number of participants, years during which the study took place, length of follow up, and main outcome measures. * Please quantify the main results (with 95% CIs and p values).

Please rephrase “we found little evidence…” to be more specific. The same goes for the sentence in the main conclusions

Limitations of this observational study must be stated more explicitly and please provide 2-3 limitations 

“supporting recent policy changes in some countries” is a bit vague and we suggest removing this or rephrasing to be more specific. 

Author Summary

Throughout: please use square brackets for references and these should be placed after all punctuation. Please format the bibliography using Vancouver style 

Introduction

I would suggest removing this sentence as it appears to be based on conjecture “All those studies grouped bouts ≥10 mins together, probably because they lacked power to explore more bouts length categories”

Suggest rephrasing this “have used two statistics…”

“Our study is the largest observational study of activity bouts to date and is the first to assess associations of MVPA” please add “to our knowledge” to temper assertions of primacy 

Methods

The link in “Analyses were performed in R version 3.5.1, Matlab r2015a or Stata version 15, and all of our analysis code is available at [https://github.com/MRCIEU/UKBActivityBoutLength” is not working, please provide a working link here and in the discussion. This link should also be provided in the data availability statement.

Results

Please provide p values along with 95% CI as needed. Please note all p-values should be exact, unless p<0.001

Discussion

Please temper associations of primacy by adding “to our knowledge” for example in Line 309

Please ensure that the study is reported according to the STROBE guideline, and include the completed STROBE checklist as Supporting Information. When completing the checklist, please use section and paragraph numbers, rather than page numbers. Please add the following statement, or similar, to the Methods: "This study is reported as per the Strengthening the Reporting of Observational Studies in Epidemiology (STROBE) guideline (S1 Checklist)." 

Did your study have a prospective protocol or analysis plan? Please state this (either way) early in the Methods section.

Comments from the reviewers:

Reviewer #1: To the Authors

You have examined the role of substituting time spent asleep, sedentary, in light and moderate-to-vigorous intensity activity (MVPA) on the associations with mortality, including examining the role of bout length of MVPA (1-15min, 16-40min, and 41+ min bouts). The substitution of time across intensities has been addressed in recent meta-analyses with more deaths and is less novel but the influence of bout length is a matter of much current debate, since some health authorities have recently changed their public health recommendations for MVPA to occur in at least 10-min bouts; it therefore seems rather odd that this paper has defined short bout durations across this divide of 10-min - this is where the uncertainty still lies and the current analysis is a lost opportunity for addressing this issue; it is vital to re-parameterise the bouts to explore the <10-min bout duration range and then compare that to the 10-min and above estimates. The standard time format of derived activity measures in UK Biobank is 5-sec bouts so there is ample opportunity to address this issue. 

Confounding by prevalent disease has not been adequately accounted for and the compositional nature of the time-bound data should be ideally complimented with analyses which account for that in a different way.

Specific comments 

Number of diseases at pre-baseline is a poor way to account for confounding by prevalent disease; you should use binary indicators of each disease as they have different impact on both activity and mortality. Moreover, as the accelerometer measurement was done about 5 years after the main study baseline, you need to use the routine health data, eg Hospital Episode Statistics which is available in UK Biobank, to reclassify disease status at the accelerometer baseline used in this analysis.

It may be worth pointing out more clearly in the confounding section that you are using age at the time of accelerometer wear (and not at main study baseline).

Seasonal adjustment of accelerometer wear is better achieved using cosine functions as previous Biobank analyses have done.

Did you calibrate the accelerometer readings to local gravity, eg using the approach described by van Hees? Raw data from this sensor is uncalibrated and calibration will greatly reduce noise and can be performed on almost all individual accelerometer files. Your flowchart suggests that you may have performed this but then again maybe not as you do not loose any data in this step.

Your hybrid approach of vector magnitude-based MVPA classification, combined with separation of the non-MVPA component into the other categories by the activity type classification method seems very appealing - do you have any evidence of its validity, though? Note also that the activity classification uses a number of accelerometer features, some of which are orientation-specific; there are two main orientations of the accelerometer measurements in UK Biobank (as two versions of the monitor was used) and possibly an additional actual orientations owing to participants turning the puck inside the wristband upside down or reverse-lateral and wearing it on left or right wrist (all of which makes it challenging to reference accelerometer measurements to the anatomical coordinates). Suggest use version of activity classification without orientation-sensitive signal features.

You collapsed the time-series to 1-min resolution of activity types before any further analysis? That is fine but you should justify this, particularly given your research question here! Note, epoch resolution and bout quantification interacts, see for example paper by Orme et al. Also note that working with 1-min data would mean that your intensity distribution estimates are different to those reported using the derived 5-sec movement intensity data available in the data showcase - again, this is fine but should be highlighted when comparing to other work.

It is fine to use 10-min increments as the unit of time substitution in the analysis of association and your definitions of sedentary bout exposures are fine too. However, you should revisit the categorization of MVPA bout durations for defining these exposures; you must have one category boundary at 10-min (below and above), and it would make sense to have at least a few in the low range where all the variance is! Given you epoch decision and current guideline debate, I would suggest one category should be 1-min, then perhaps 2-9 min, then 10-15min and the remaining ones you already have (although I would probably collapse those if it was me). Your downstream analytical approach requires these to be mutually exclusive but it would actually be better to think about them as nested variables; at least 1-min duration, at least 5-min duration, at least 10-min duration, and so on - please reflect and respond.

You have conducted a time compositional analysis of a bounded phenomenon (24 hours in a day), which makes assumptions about linearity in a constrained variance space; consider supplementing your time substitution analysis with a compositional data analysis or multivariate pattern analysis.

Mortality results have been updated in 2020 and you should therefore rerun your analysis for greater power. Suggest include sensitivity analysis excluding those who died within the first year or two to account for potential reverse causality / confounding by ill-health (I think the 5 years you are mentioning in passing in the discussion is a bit extreme; this has been suggested by those who do not understand regression dilution).

Your findings do NOT directly support the abolition of the 10-min MVPA bout requirement as you have not compared associations for <10-min bout MVPA with those for 10-min or longer bouts!

Previous work on time substitution in Biobank showed broadly similar mortality associations using the self-reported variables - maybe worth comparing your results to?

Reviewer #2: This is a nice study examining associations between physical activity and mortality. The study includes a large sample of UK adults (~80k), measured with accelerometry. This is among the largest studies that include accelerometry in a sample of adults. I do have a few concerns, perhaps the most relevant is the short time of follow-up.

Major:

1 - Participants were assessed in 2013-2015 and have follow-up through 2018. Follow-up time is expected to range from 3-5 years which can be a concern. It is not clear how limited follow-up might have impacted the results but it would be helpful to see sensitivity analyses for England/Wales vs Scotland, which seem to have different follow-up times and also after excluding first 2-3 years of follow-up. It would also help if the authors include a sensitivity analysis excluding individuals with poor health or with major chronic conditions.

2 - The study also includes a rather limited set of confounders. Important confounders such as diet and physical mobility limitations were not included. These are important confounders that can add beyond the ones that were included (eg, BMI). This issue of unmeasured confounding is problematic, particularly given the relatively weak associations that were found for MVPA-mortality. 

Minor:

1 - Include follow-up information earlier in the paper (in the all-cause mortality section). 

2 - Is there any proxy for mobility limitation that could be used as an additional confounder?

3 - About the MVPA bouts: I was expecting to see bouts of 1-10 min; 11+, etc. The intro (appropriately) address the discussion of 10+ bouts but the categorization used in the paper (15+) is not aligned with this bout criteria.

Also, after looking at the bout distributions, looks like there is not much data for longer bouts. The 1-15min bouts is a pretty long bout - it represents the majority of total MVPA (almost 90% by looking at table S2). This category includes non-bouted and bouted PA (to a max of 15min) given that the lowest resolution is 1-min epochs. Non-bouted and bouted activity should be separated here. It would seem more reasonable to use 1-4, 5-9, 10+, etc. or 1-9, 10+. Also, were interruptions allowed when creating these bouts? 

The longer bouts (16min +) is likely skewed and makes me doubt there is enough data here to test these associations. How many people reported PA in each of the bout levels? Showing these distributions and amount of '0s" will tell how much data there is for this particular analysis. 

Finally, still related to bouts - looks there is pretty much no data on 41+ bouts of PA. Pease show the distribution for this as well. 

4 - Include an explanation for using age as time metric as oppose to time of follow-up. I wondered if truncating birth at July 1st results in losing the benefit of using age as time metric. 

5- Please include a Table 1 with information on demographics and follow-up time. This in the suppl material but it would be helpful to see this in the main paper. Also, Table S1 shows there were 538 events which conflicts with ~700 reported in the main text. Please clarify.

6 - In the discussion section, references in support of excluding the bout criteria (as per old guidelines recommendation) can't be supported here as bouts were not generated to test this assumption. See earlier comment and refine bout classifications. 

7 - The following statement shows up a couple times in the paper but its not accurate:

"There are no previous studies assessing the association of MVPA bout length with mortality"

Here is a study that should be cited/discussed for this purpose:

Moderate‐to‐Vigorous Physical Activity and All‐Cause Mortality: Do Bouts Matter? 

Saint-Maurice PF et al. JAHA 2018

8 - I mentioned this before but suggest that the discussion on short follow-up be expanded as a critical limitation. I also suggest additional sensitivity analyses. Eg, excluding people with chronic conditions. 

The following paper might be helpful for this purpose:

https://pubmed.ncbi.nlm.nih.gov/32472927/

Given the weak associations with MVPA, a small degree of confounding is likely to attenuate these effects to become null. This should be part of the discussion here as well. 

Reviewer #3: I confine my remarks to statistical aspects of this paper. These were very well done, but I have a few questions and comments before I can recommend publication.

First, I wish more of the technical details were in the main paper, but I suppose that is a matter for the editors; it isn't a mistake to put it in a supplement.

Line 160-161 I am curious about how sleep was distinguished from sedentary activity. Even if the technical details are given in an appendix, I think a plain language summary of this would be useful; after all, people do move in their sleep and some people lie still (but awake) for some time before sleeping. In fact, a plain language summary of all the levels would be useful.

Line 165-168 

 1. What was the "gold standard"? That is, you say some periods were misclassified. But how was the right classification determined? And, if the right category could be determined, why wasn't it used? It seems to me there are going to be some murky periods no matter what you do. (e.g. time spent just before sleep; time spent going to the bathroom in the middle of the night; activity that is on the border between moderate and light; and so on.

 2. Given that you state some of the misclassification (58% of MPVA) I'd suggest giving all the results, for both models.

Also, as a statistician, I'm just curious why three different programs were used --- it's fine to do so and all the programs are good ones, I'm just wondering

Overall a very interesting analysis!

Peter Flom

[LINK]

---

## [Decision Letter · Decision Letter 2]

3 Dec 2020

Dear Dr. Millard,

Thank you very much for submitting your revised manuscript "Association of changing physical activity intensity and bout length with mortality: a prospective study of 79,503 participants in UK Biobank" (PMEDICINE-D-20-02260R2) for consideration at PLOS Medicine. 

The revisions were seen again by the previous reviewers, whose comments are enclosed below; I hope you find them constructive. Two now recommend acceptance but one reviewer (r#1), although acknowledging the improvements made, does still ask for further clarifications on the methods and reporting. At this stage we'd ask that you respond to those points in a further revision before we can make a final decision on the paper. Please note this reviewer says there are some errors in internal links in the document; I checked this and although these ("error!" links) appear in the compiled pdf, they seem to be OK in the submitted Word file. The editors are fine for the authors to include a mention that preplanned analyses were modified due to input obtained during peer review. 

The reviews are appended at the bottom of this email and any accompanying reviewer attachments can be seen via the link below:

[LINK]

We expect to receive your revised manuscript by Dec 24 2020 11:59PM. Please email us (plosmedicine@plos.org) if you have any questions or concerns.

We look forward to receiving your revised manuscript. 

Sincerely,

Emma Veitch, PhD

PLOS Medicine

On behalf of Adya Misra, PhD, Senior Editor, 

PLOS Medicine

plosmedicine.org

*We'd recommend that the data availability statement provided in the article submission system is updated to include the github links for code availability (provided in the authors' response and main text). 

Comments from the reviewers:

Reviewer #1: 

The revisions have improved this paper substantially, particularly the update of follow-up and the re-specification of bouts around the currently discussed bout duration boundary of 10 minutes. There are, however, a few outstanding concerns remaining as outlined below.

Title: You have not assessed change in physical activity but between-individual differences - change title accordingly (eg, delete "changing").

Abstract:

It is not a trial so your conclusion should be that activity is associated with lower mortality, not "reduced" mortality. Same issue with "increased" mortality risk - it should read "higher". Make global check for causal language.

Introduction:

There are other papers from the cohort reporting on the general association between accelerometry-measured exposure and mortality. For example, a recent paper by Strain et al (Nature Medicine, 2020) reported on the associations between accelerometry-measured activity and mortality using the same dataset but did not look at the potential importance of bouts - suggest reword intro slightly to leap from there. 

Note that the recently launched WHO guidelines for physical activity have also lost the 10-min bout requirement, and there is increased recognition of light activity too - may allow you a slightly elevated pitch to refer to global guidelines rather than just US/UK national?

Methods: 

Invitation to join the accelerometry subsample was not random; everyone with a valid email in the cohort were invited, except those currently taking part in substudies.

Thanks for confirming that you have calibrated your accelerometer data to gravity; they would otherwise not have been valid and we could stop right there! Please include appropriate citation for the method - the Biobank tool uses the van Hees method (with temperature correction) and other tools use either that or the Lukowic method, both of which are sufficient but you do have to use one.

To clarify the point about accelerometer axis orientation: All accelerometers used were Axivity AX3 but there are two versions of that. The first batch of accelerometers used in UKB had the X and the Y axes swopped around in the casing, compared to subsequent batches used. There are no other differences as the chip is the same; it simply means a rotation of 90 degrees on the wrist mount. The meta data of the .cwa files includes this information through its serial numbers but you can also see it in the data as the axis angles (pitch and roll) with gravity (low-frequency component in the data) differ between the two batches. The activity classification work by Willett et al (Sci Rep 2018 - the correct citation for the method used in Doherty et al, 2018) used the orientation of the X and Y axes of the more recent batch, so is directly applicable to that part of the UKB data but not directly to the data collected with the first batch of monitors. Although only some of the several signal features used in the activity classification method are orientation-sensitive, the optimal application of the method requires remapping X acceleration to Y and vice versa as an initial step for those who were measured with the earliest version of the accelerometer.

The splitting of the MVPA exposure category around the 10-min boundary is excellent and will make the results of this analysis far more relevant to the current debate on the issue. I have to say, the other categories do not seem entirely logical to me, eg why split MVPA at 40 minutes and not 30 minutes? And why not split the sub-10 category further, eg at 5-min, as that is where all the behavioural variance is (and one notes that exact example of 5-min bouts in your summary statement)? And why insist on alignment of sedentary and MVPA bout durations when the notion of short and long are materially different for such behaviours?

I don`t think you need to keep saying "at the request of the Reviewers" in the Methods as you have already stated it when describing deviations from your analysis plan but I refer to the Editor here for specific journal policy. 

With respect to the approach of defining exposure as nested vs mutually exclusive, I appreciate the opportunity to do the reflections for you since you ask; it is certainly not wrong to conduct a mutually exclusive categorization analysis as you have done but given that the recent debate was not about which bout duration activity should have (short or long) to confer health benefits but rather if it should have a minimum duration of 10min or not, the analysis that more directly addresses that issue is one that contrast associations of exposures with and one without a minimum bout duration definition (and not a maximum one).

Results:

Main table 1 doubles up as a drop-out analysis - fine although I would have preferred a clean main table of the data in the second column and then this one in supplement.

I see no more main tables? Are supplementary table S3a and S4 not the most essential set of results? Or do figure 2 and 3 serve this function as main results? We are not helped by the formatting issue here - there is a dead-end bookmark in my review copy. 

Discussion:

Suggest delete "uniquely" from opening statement - it reads very odd and probably isn`t even true unless you caveat it somehow but that would make it worse.

Suggest make reference to global public health recommendations by the WHO instead - seems more convincing. It is not true that the removal of 10-min MVPA bout duration requirements from US and UK guidelines were purely based on trial evidence - observational epidemiological evidence such as the type you report here was also considered, including some of the papers you cite here yourself - please rewrite.

Your points about the limitations of self-report are well made - in fact so well made that you should include them in your paper. The fact remains though that the overall conclusions about time in different intensities do align well with previous reports from the full 500k participants in the cohort with longer follow-up. 

With respect to the proposed text edits on the accelerometer orientation issue, it is wrong to blame this on the participants as those in the early batch most likely did what they were told to do with that version of the monitor (so they did not wear it incorrectly) - please rephrase, including making the point that your hybrid approach of using vector-magnitude-based MVPA mitigates against this being a major issue for most of your main analysis (as vector magnitude is not orientation-specific).

"… any true effect of activity on mortality being short-lived" - I can`t quite decide whether this is an unfortunate phrasing of words or rather smart or funny but if this was unintentional, you should probably rephrase it!

An issue which requires further discussion is the fact that 1-min epoch data are 1-min bouts - maybe just say exactly that where you mention that it might change if you had used another epoch setting which is a bit vague.

Also, whilst I agree that some previous analyses have not accounted for overall time in sedentary or MVPA, you have not accounted for intrinsic differences in the intensity of MVPA between bouts; it is perfectly possible that those who go for longer MVPA bouts are also those who run faster when doing MVPA! In the recent Strain paper from the cohort, total volume was a main driver of the association with mortality, with additional benefit suggested from increased fraction of that volume coming from MVPA. Your analysis does not account for volume differences and intensity differences within exposure categories (which also makes your cutpoint decision far more influential) but the previous analysis does - a paragraph to discuss this issue should suffice.

General formatting errors throughout for references to within-document items, eg figs/tables.

Reviewer #2: I appreciate the authors effort to address my concerns. Well done! 

Reviewer #3: The authors have addressed my concerns and I now recommend publication

Peter Flom

[LINK]

---

## [Editor Report · Decision Letter 3]

15 Jul 2021

Dear Dr. Millard,

Thank you very much for re-submitting your manuscript "Association of physical activity intensity and bout length with mortality: a prospective study of 79,503 participants in UK Biobank" (PMEDICINE-D-20-02260R3) for consideration at PLOS Medicine. We do apologize for the long delay in our response. 

I have discussed the paper with editorial colleagues and our academic editor, and I am pleased to tell you that, once the remaining editorial and production issues are fully dealt with, we expect to be able to accept the paper for publication in the journal.

[LINK]

Please let me know if you have any questions, and we look forward to receiving the revised manuscript.

Sincerely,

Richard Turner PhD

rturner@plos.org

Requests from Editors:

Please adapt the data statement to: "... available from UK Biobank for researchers who meet the criteria for access to confidential data ..." or similar. 

Please remove the word "prospective" from the title (we think that your study is a retrospective analysis of prospectively gathered data). So as to comply with journal style we suggest: "Physical activity intensity and bout length and mortality in 79,503 UK Biobank participants: a population-based cohort study".

At line 22, should that be "... less light activity ..."?

At line 24 and elsewhere, we suggest making that "sedentary time".

In the abstract, we suggest quoting the 95% CI and p values immediately after the point estimates, e.g., "... (HR 1.02 [95% CI 1.01 to 1.02, p<0.001] per 10 minutes more sedentary time ...)".

At line 28, we suggest removing "UK Biobank had a 5% recruitment response", as this can be mentioned in the main text.

At line 32 please make that "was associated" as you are reporting findings, with the surrounding wording adapted to be consistent with this. 

At line 82, for example, please adapt the reference call-out to the style: "... amounts alone [4,5]." (noting the absence of spaces within the square bracket). 

Please restructure the paragraph beginning at line 109, where the elements of discussion should be removed or transferred to the Discussion section. We anticipate 1-2 sentences stating the aim and, to an extent, the methodology of your study. The sentence beginning at line 118 should be removed. 

You mention an analysis plan at line 124. Please include this as an attachment if available, referred to in the text. 

At line 164 and elsewhere as appropriate, please use the style "follow-up".

We ask you to adopt more circumspect language in places. For example, at line 385 to avoid over-generalizing we suggest amending the wording to: "In this study, we found that time spent in MVPA was associated with lower mortality ..." or similar (with the tenses adjusted to match in the rest of the paragraph). 

Please use the journal name abbreviation "PLoS ONE" in the reference list.

Please add "[preprint]" to any preprints cited, reference 29 perhaps being one.

Please remove the information on funding and the UK Biobank application from the end of the text. The former will appear in the article metadata in the event of publication, via entries in the submission form, and the latter can be moved to the Methods section. 

***

---

## [Editor Report · Decision Letter 4]

27 Jul 2021

Dear Dr. Millard,

Thank you very much for re-submitting your manuscript "Association of physical activity intensity and bout length with mortality: an observational study of 79,503 UK Biobank participants" (PMEDICINE-D-20-02260R4) for consideration at PLOS Medicine.

Following further discussions among the editors, we will need to ask you to respond to some minor comments from our academic editor before we are in a position to proceed further. The remaining issues that need to be addressed are listed at the end of this email: please take these into account before resubmitting your manuscript.

Please let me know if you have any questions, and we look forward to receiving the revised manuscript.   

Sincerely,

Richard Turner, PhD

rturner@plos.org

Requests from Editors:

Does "income (pounds)" need to be specified in table 1?

Comments from Academic editor:

I note an issue with supplementary figures S10-12 which show the compositional analysis results (judged by some to be the correct way to examine the main question posed in this paper, so fairly essential). Specifically, the authors need to explain what the two curves in each colour or panel represents, and secondly comment on what the results actually show; the all-too-brief statement in the manuscript says that that these analyses show the same as the main results but in some cases they appear to show the exact opposite or indeed very complex U-shaped associations! This needs a proper description and a proper discussion.

A minor point is to sharpen up and strengthen the statement in the summary of “what do these findings mean”: The authors have analysed the accelerometer data in 1-min time resolution, and so the shortest bout of any activity category is 1 minute. Although it is not directly reported anywhere (but perhaps should be), we can deduce that the most common “short” MVPA bout is very close to 1 minute, given that the median time spent in total MVPA is 92 min per day and the median time spent in short MVPA bouts is 72 min per day. Hence, the example of 5-min bout duration for MVPA is a bit odd and probably wrong or at least sending the wrong message; what we have learned from this analysis is that MVPA bouts as short as 1 minute in duration have benefits for human health – a more accurate and more powerful message, providing very strong support for the most recent health recommendations.

***

---

## [Editor Report · Decision Letter 5]

5 Aug 2021

Dear Dr Millard, 

On behalf of my colleagues and the Academic Editor, Dr Brage, I am pleased to inform you that we have agreed to publish your manuscript "Association of physical activity intensity and bout length with mortality: an observational study of 79,503 UK Biobank participants" (PMEDICINE-D-20-02260R5) in PLOS Medicine.

PRESS

Sincerely, 

Richard Turner, PhD 

rturner@plos.org