# Supplementary material for: Association of physical activity intensity and bout length with mortality: An observational study of 79,503 UK Biobank participants
Source: PLoS Med. 2021 Sep 15;18(9):e1003757. doi: 10.1371/journal.pmed.1003757 (PMC8480840; doi:10.1371/journal.pmed.1003757)
Supplement: S1 Fig — (PDF) [file pmed.1003757.s002.pdf]

S1 Fig. Directed acyclic graph illustrating path between bout length and survival through a common cause of bout length and total time spent sedentary

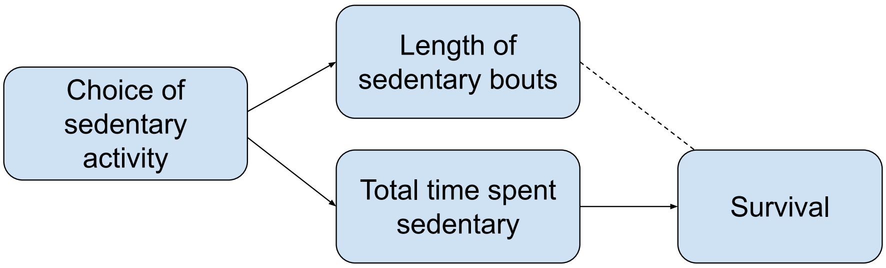

In this illustration total time spent sedentary is the only direct cause of survival. A person's choice of sedentary activity is a common cause of both sedentary bout length and total time spent sedentary. The length of sedentary bouts is not a determinant of survival, but there is an unblocked path between this variable and survival, via 'choice of sedentary activity' as this variable is a confounder (DAG 'fork') between sedentary bout length and total time spent sedentary that is not conditioned upon [9]. This induces a correlation between bout length and survival (denoted by dashed line connecting them).

In this example, an unbiased estimate of the effect of sedentary bout length on survival can be obtained by adjusting for 'choice of sedentary activity' (or in general all common causes of sedentary bout length and total time spent sedentary) or by adjusting for total time spent sedentary – both these approaches would block the non-causal path between sedentary bout length and survival [9].
