# Supplementary material for: Association of physical activity intensity and bout length with mortality: An observational study of 79,503 UK Biobank participants
Source: PLoS Med. 2021 Sep 15;18(9):e1003757. doi: 10.1371/journal.pmed.1003757 (PMC8480840; doi:10.1371/journal.pmed.1003757)
Supplement: S2 Fig — (PDF) [file pmed.1003757.s003.pdf]

S2 Fig. Directed acyclic graph illustrating hypothesised confounding factors and potential mediators

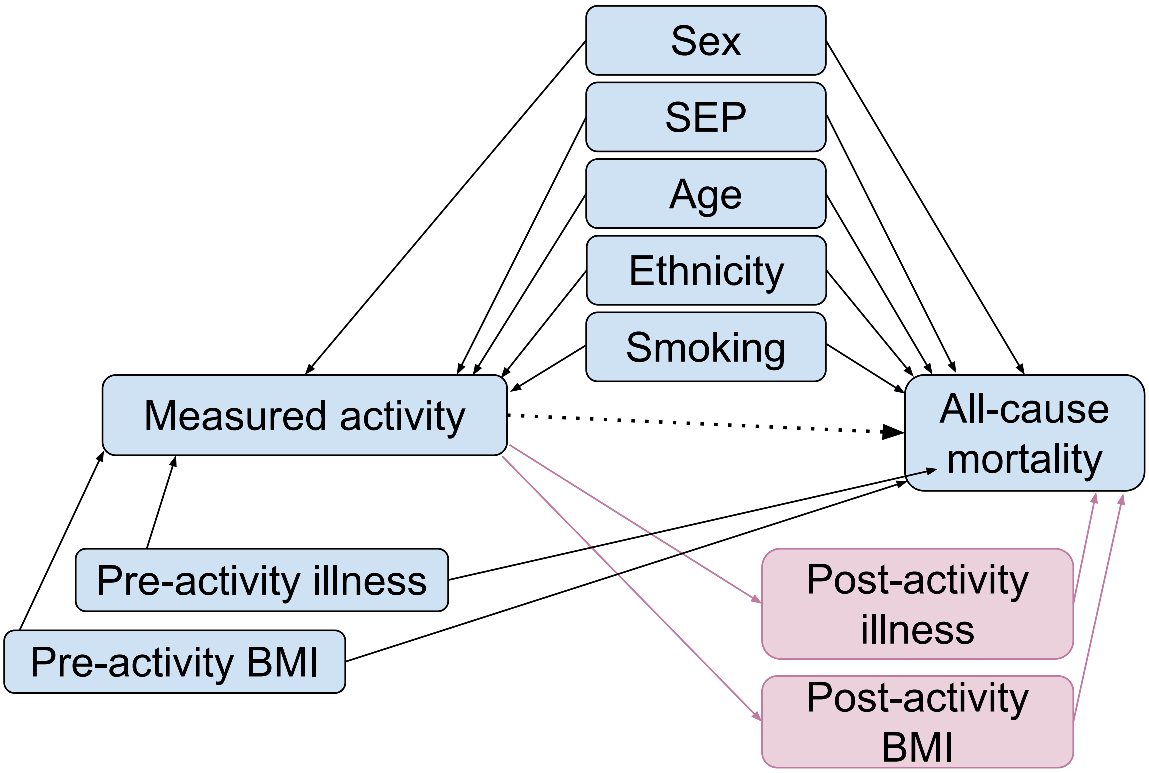

SEP: socio-economic position; BMI: body mass index.

Sex, age and ethnicity cannot be affected by activity so cannot be on the causal pathway between activity and all-cause mortality.

BMI has been shown to affect physical activity [11], and is also likely to be on the causal pathway between activity and mortality. Ill-health is also likely to both confound the association between activity and mortality, and be on the causal pathway from activity to mortality.
