# Supplementary material for: Association of physical activity intensity and bout length with mortality: An observational study of 79,503 UK Biobank participants
Source: PLoS Med. 2021 Sep 15;18(9):e1003757. doi: 10.1371/journal.pmed.1003757 (PMC8480840; doi:10.1371/journal.pmed.1003757)

S3 Fig. Distributions of the number of valid days of accelerometer data for participants included in our samples

a) Complete days version

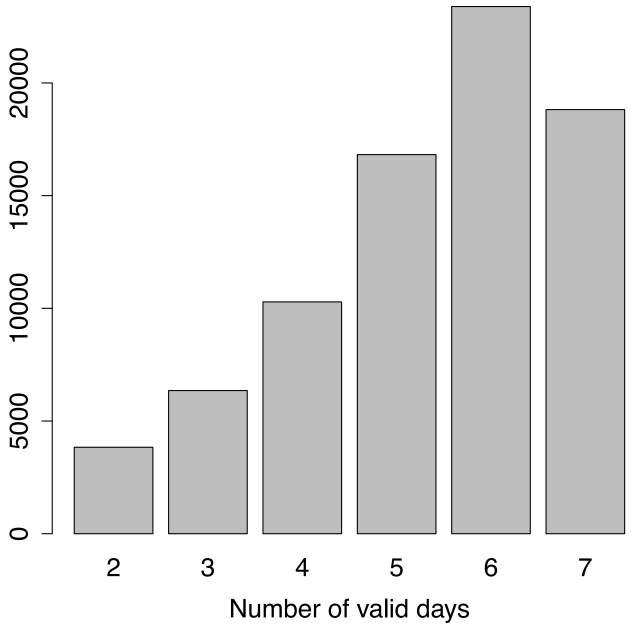

b) Other day imputed version

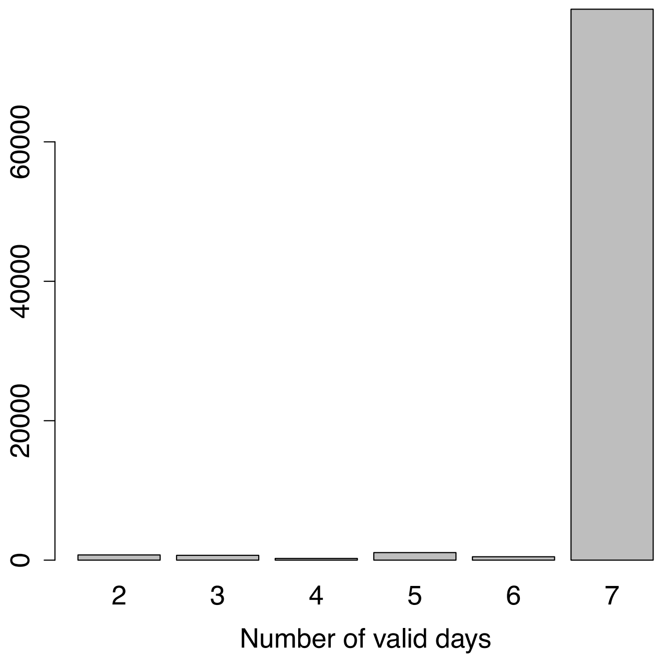

Supplement: S3 Fig — (PDF) [file pmed.1003757.s004.pdf]
