# Supplementary material for: Association of physical activity intensity and bout length with mortality: An observational study of 79,503 UK Biobank participants
Source: PLoS Med. 2021 Sep 15;18(9):e1003757. doi: 10.1371/journal.pmed.1003757 (PMC8480840; doi:10.1371/journal.pmed.1003757)
Supplement: S6 Fig — (PDF) [file pmed.1003757.s007.pdf]

S6 Fig. Association of lower amounts of time spent in baseline activity category coupled with higher amounts of time in comparison category, with all-cause mortality using 'other day' imputed accelerometer data

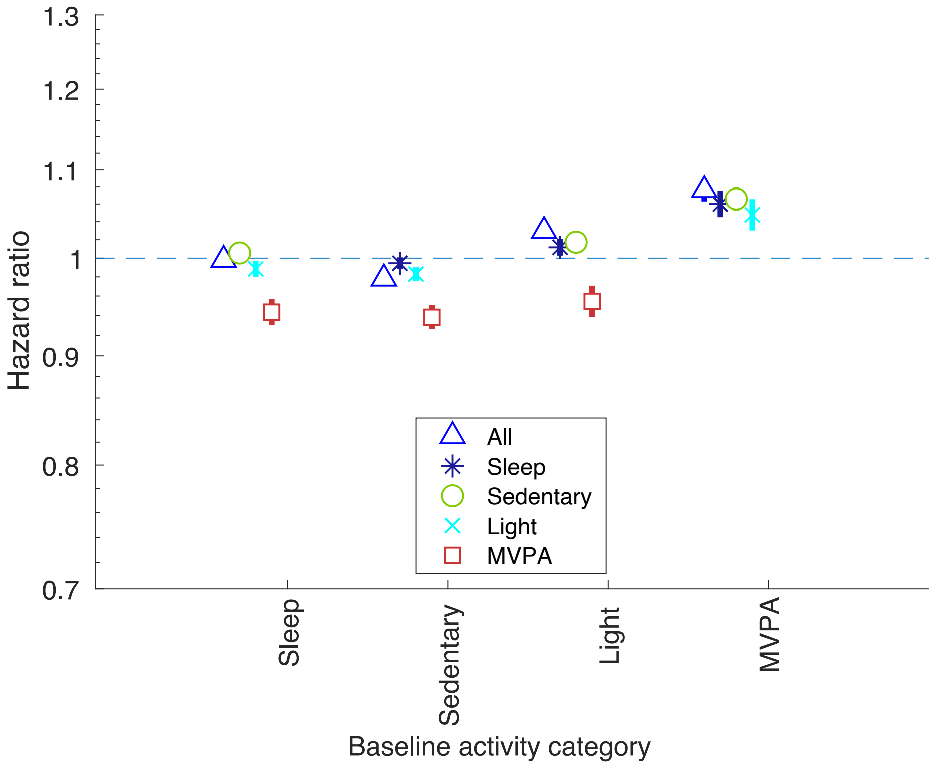

Hazard ratio of spending 10 minutes more time on average per day in comparison activity category, coupled with spending 10 minutes less time in baseline activity category. Plotted data are reported in S4 Table. Using the 'other day' imputed data. Equivalent results using the complete days approach are shown in Figure 2 in the main paper.

Covariates: age at accelerometer wear, sex, ethnicity, season, smoking, SEP (education, Townsend deprivation index, income), BMI, and three indicators denoting whether the participant had cardiovascular disease, cancer or respiratory disease prior to accelerometer wear.
