# Supplementary material for: Association of physical activity intensity and bout length with mortality: An observational study of 79,503 UK Biobank participants
Source: PLoS Med. 2021 Sep 15;18(9):e1003757. doi: 10.1371/journal.pmed.1003757 (PMC8480840; doi:10.1371/journal.pmed.1003757)
Supplement: S8 Fig — MVPA, moderate-vigorous physical activity. (PDF) [file pmed.1003757.s009.pdf]

S8 Fig. Results of sensitivity analysis starting follow-up one and two years after accelerometer wear: Association of time spent in MVPA and sedentary bouts of a given length, with all-cause mortality

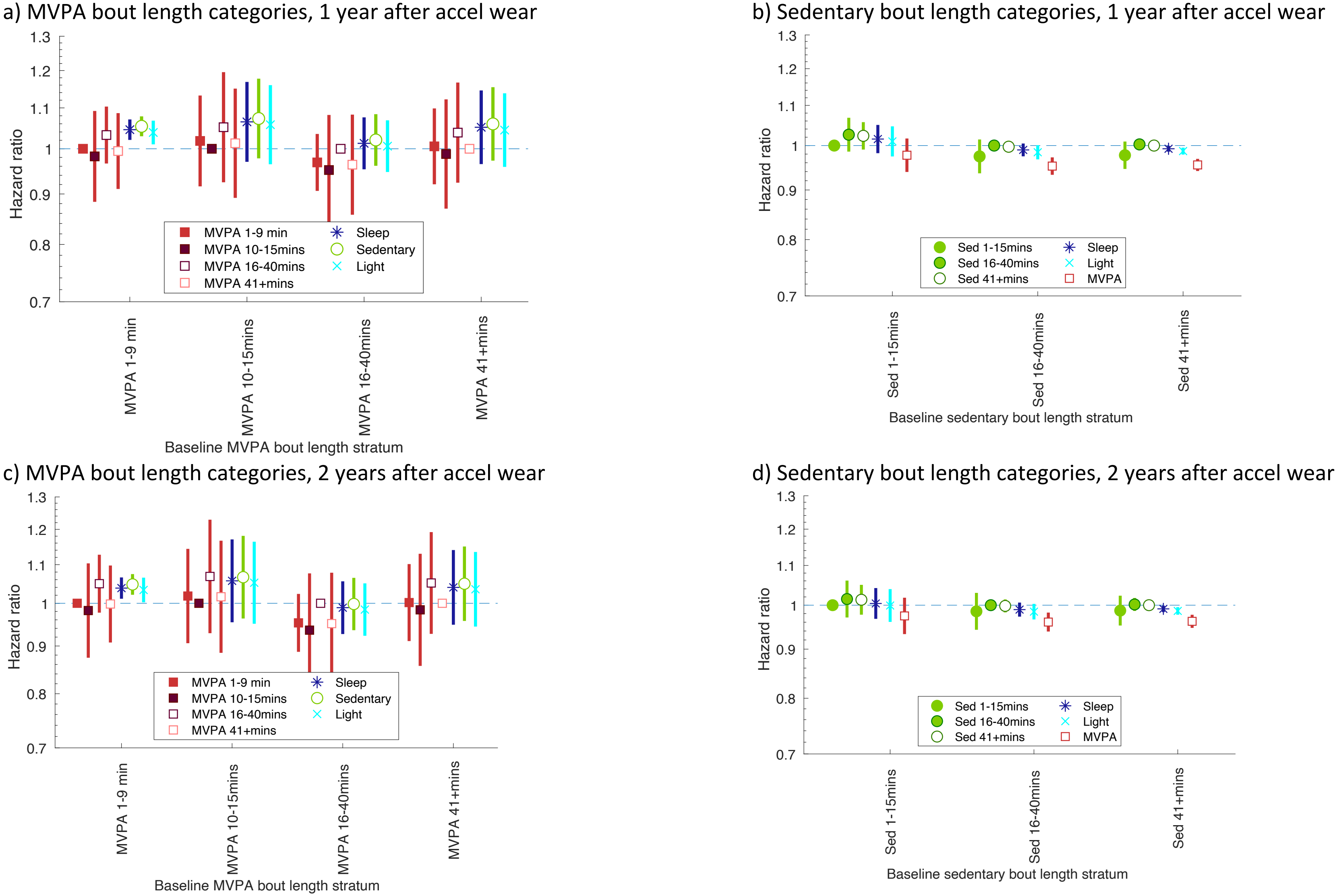

Analysis conducted using complete days data.

Hazard ratio of spending 10 minutes more time on average per day in comparison activity category, coupled with spending 10 minutes less time in baseline activity category.

Covariates: age at accelerometer wear, sex, ethnicity, season, smoking, SEP (education, Townsend deprivation index, income), BMI, and three indicators denoting whether the participant had cardiovascular disease, cancer or respiratory disease prior to accelerometer wear.
