# Supplementary material for: Association of physical activity intensity and bout length with mortality: An observational study of 79,503 UK Biobank participants
Source: PLoS Med. 2021 Sep 15;18(9):e1003757. doi: 10.1371/journal.pmed.1003757 (PMC8480840; doi:10.1371/journal.pmed.1003757)
Supplement: S10 Fig — (PDF) [file pmed.1003757.s011.pdf]

S10 Fig. Results of sensitivity analysis using isometric log-ratio transformed activity variables: Association of time spent in activity categories, with all-cause mortality

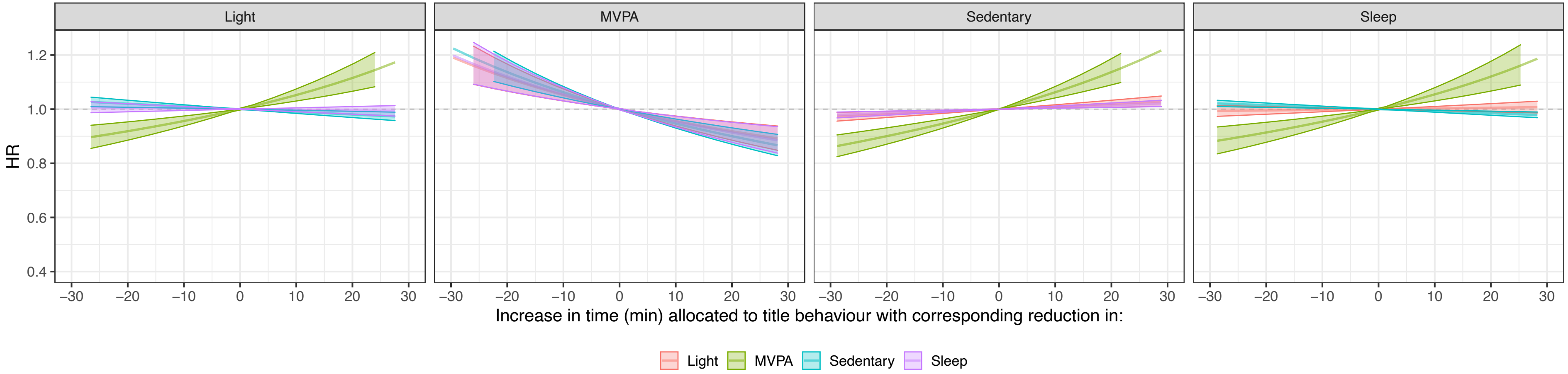

Each curve shows the hazard ratio (and corresponding 95% confidence interval) for removing the number of minutes specified on the x-axis, from the baseline category (specified in legend), and adding this amount of time to the comparison category (specified in the title of each plot). For example, the green line of the 'sleep' plot shows, for the positive values on the x-axis, the hazard ratio for less time in MVPA, coupled with spending more time sleeping. The linear relationship shown in this plot suggests that the association for doing e.g. 10 minutes less MVPA coupled with 10 minutes more sleeping is approximately half the association for doing 20 minutes less MVPA coupled with 20 minutes more sleeping.

Analysis conducted using complete days data.

Covariates: age at accelerometer wear, sex, ethnicity, season, smoking, SEP (education, Townsend deprivation index, income), BMI, and three indicators denoting whether the participant had cardiovascular disease, cancer or respiratory disease prior to accelerometer wear.
