# Supplementary material for: Association of physical activity intensity and bout length with mortality: An observational study of 79,503 UK Biobank participants
Source: PLoS Med. 2021 Sep 15;18(9):e1003757. doi: 10.1371/journal.pmed.1003757 (PMC8480840; doi:10.1371/journal.pmed.1003757)
Supplement: S11 Fig — MVPA, moderate-vigorous physical activity. (PDF) [file pmed.1003757.s012.pdf]

S11 Fig. Results of sensitivity analysis using isometric log-ratio transformed activity variables: Association of time spent in MVPA bouts of a given length, with all-cause mortality

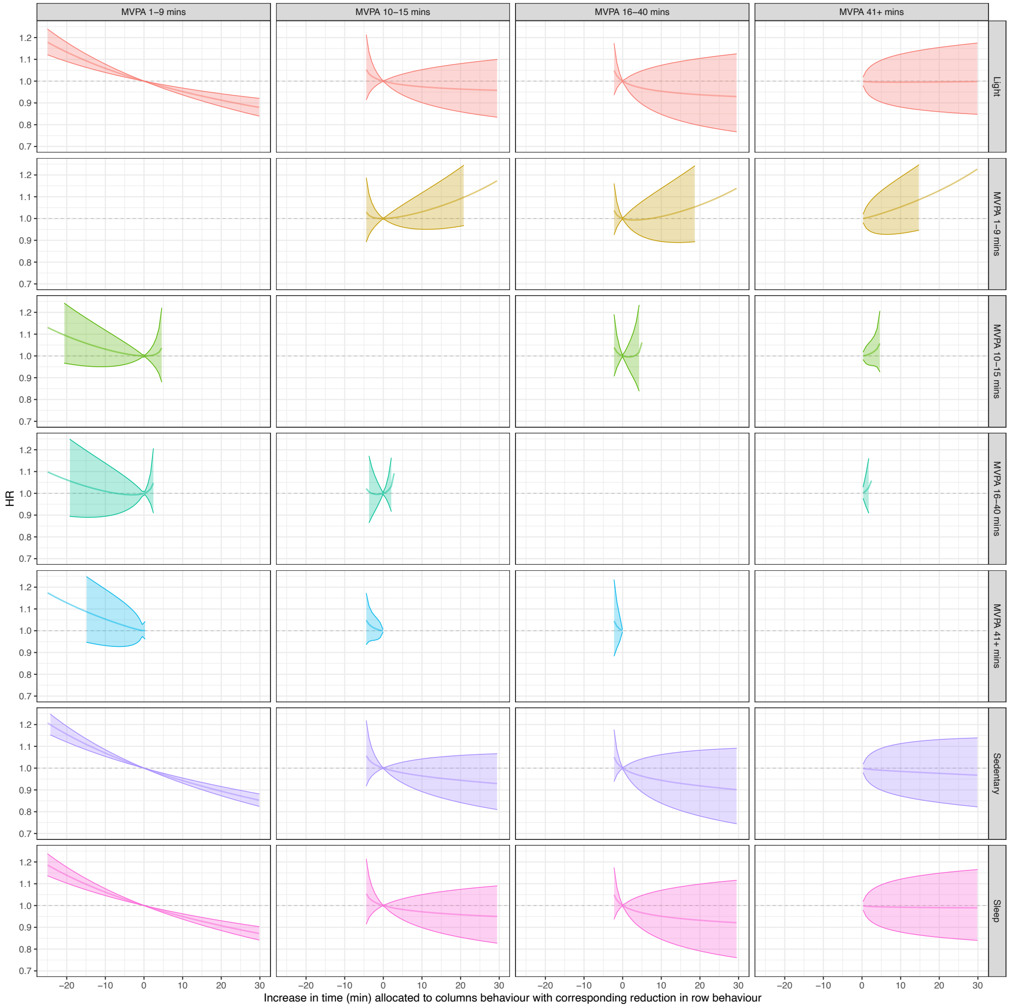

Each curve shows the hazard ratio (and corresponding 95% confidence interval) for removing the number of minutes specified on the x-axis, from the baseline category (specified in row titles), and adding this amount of time to the comparison category (specified in the column titles). For example, the curve for MVPA 1-9 mins versus sleep shows, for the positive values on the x-axis, the hazard ratio for more time spent in very short MVPA bouts, coupled with less time spent sleeping.

Analysis conducted using complete days data.

Covariates: age at accelerometer wear, sex, ethnicity, season, smoking, SEP (education, Townsend deprivation index, income), BMI, and three indicators denoting whether the participant had cardiovascular disease, cancer or respiratory disease prior to accelerometer wear.
