# Supplementary material for: Association of physical activity intensity and bout length with mortality: An observational study of 79,503 UK Biobank participants
Source: PLoS Med. 2021 Sep 15;18(9):e1003757. doi: 10.1371/journal.pmed.1003757 (PMC8480840; doi:10.1371/journal.pmed.1003757)
Supplement: S12 Fig — (PDF) [file pmed.1003757.s013.pdf]

S12 Fig. Results of sensitivity analysis using isometric log-ratio transformed activity variables: Association of time spent in sedentary bouts of a given length, with all-cause mortality

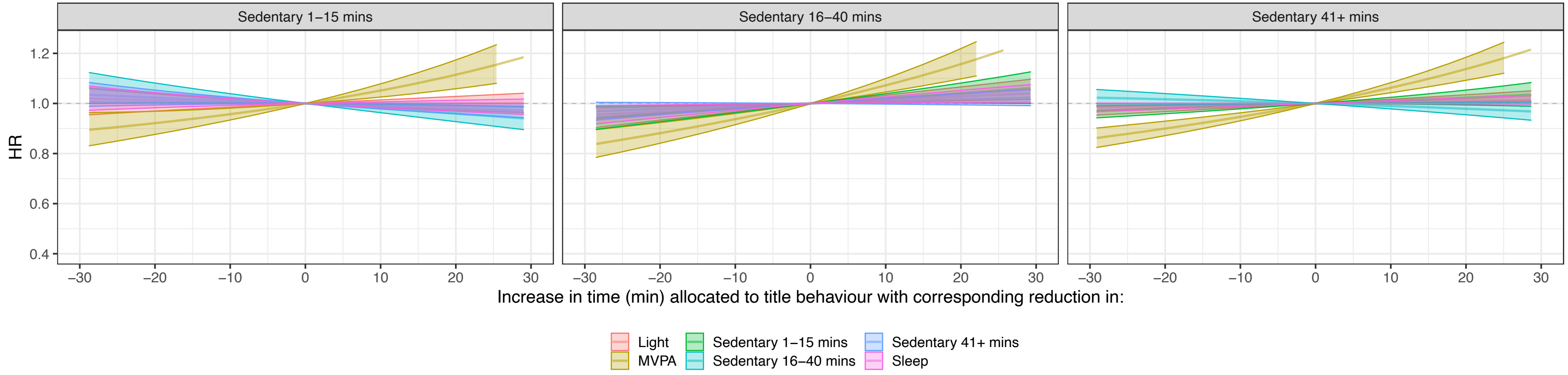

Each curve shows the hazard ratio (and corresponding 95% confidence interval) for removing the number of minutes specified on the x-axis, from the baseline category (specified in legend), and adding this amount of time to the comparison category (specified in the title of each plot). For example, the yellow line of the 'Sedentary 41+mins' plot shows, for the positive values on the x-axis, the hazard ratio for less time in MVPA, coupled with spending more time in sedentary bouts of duration 41 minutes or longer. The linear relationship shown in this plot suggests that the association for doing e.g. 10 minutes less MVPA coupled with 10 minutes more time spent in long sedentary bouts is approximately half the association for doing 20 minutes less MVPA coupled with 20 minutes more time spent in long sedentary bouts.

Analysis conducted using complete days data.

Covariates: age at accelerometer wear, sex, ethnicity, season, smoking, SEP (education, Townsend deprivation index, income), BMI, and three indicators denoting whether the participant had cardiovascular disease, cancer or respiratory disease prior to accelerometer wear.
