# Supplementary material for: Association of physical activity intensity and bout length with mortality: An observational study of 79,503 UK Biobank participants
Source: PLoS Med. 2021 Sep 15;18(9):e1003757. doi: 10.1371/journal.pmed.1003757 (PMC8480840; doi:10.1371/journal.pmed.1003757)
Supplement: S13 Fig — (PDF) [file pmed.1003757.s014.pdf]

S13 Fig. Directed acyclic graph illustrating the potential for collider bias due to selection into the study sample

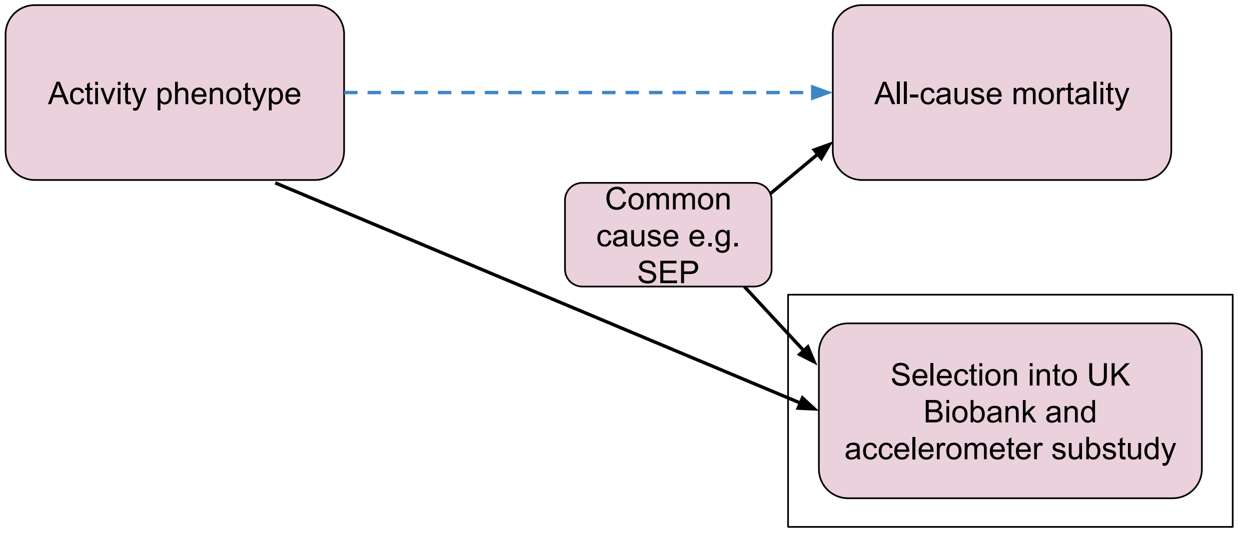

The blue dashed arrow indicates the hypothesised association we are testing.

Solid black arrows indicate an alternative pathway due to conditioning on selection into the study (indicated by a square box surrounding this node), which may bias estimates of the hypothesised association.
