# Supplementary material for: Association of physical activity intensity and bout length with mortality: An observational study of 79,503 UK Biobank participants
Source: PLoS Med. 2021 Sep 15;18(9):e1003757. doi: 10.1371/journal.pmed.1003757 (PMC8480840; doi:10.1371/journal.pmed.1003757)
Supplement: S1 Table — (DOCX) [file pmed.1003757.s015.docx]

S1 Table: Summary statistics of UK Biobank participants who are in our sample, compared with those who were invited to wear an accelerometer but are not in our sample, for complete days version

|  | **Invited to wear accelerometer but not in our sample** | | **Has accelerometer data and in our sample** | | **Difference between participants in our sample and those invited but not in our sample** |
| --- | --- | --- | --- | --- | --- |
|  | **Number of participants** | **Mean (SD) or N (%)** ^a^ | **Number of participants** | **Mean (SD) or N (%)** ^a^ | **Odds ratio [95% CI]** ^b^ |
| Age in years at assessment centre (years) | 156,953 | 56.00 (8.06) | 79,503 | 55.89 (7.83) | 0.998 [0.997, 0.999] |
| Sex - % male | 156,953 | 71,792 (45.74) | 79,503 | 36,196 (45.53) | 0.991 [0.975, 1.009] |
| Ethnicity – white | 156,032 | 147,458 (94.50) | 79,503 | 77,145 (97.03) | *baseline* |
| Black or black British |  | 2,410 (1.54) |  | 654 (0.82) | 0.519 [0.476, 0.566] |
| Asian or Asian British |  | 3,125 (2.00) |  | 686 (0.86) | 0.420 [0.386, 0.456] |
| Other |  | 3,039 (1.95) |  | 1018 (1.28) | 0.640 [0.596, 0.688] |
| Smoking status - % ever | 156,122 | 69,130 (44.28) | 79,503 | 34,270 (43.11) | 0.953 [0.937, 0.970] |
| Income – less than 18,000 | 128,897 | 21,794 16.91) | 79,503 | 11,557 (14.54) | 1.075 [1.067, 1.083] |
| 18,000 - 30,999 |  | 32,325 (25.08) |  | 19,197 (24.15) |  |
| 31,000 – 51,999 |  | 36,866 (28.60) |  | 22,864 (28.76) |  |
| 52,000 – 100,000 |  | 29,702 (23.04) |  | 20,063 (25.24) |  |
| >100,000 |  | 8,210 (6.37) |  | 5,822 (7.32) |  |
| BMI | 155,998 | 27.32 (4.70) | 79,503 | 26.71 (4.52) | 0.971 [0.970, 0.973] |
| Respiratory disease diagnosis ^c^ | NA  *156,953* | NA  *63,107 (40.20)* | 79,503  *79,503* | 32,837 (41.30)  *31,832 (40.04)* | NA  *0.993 [0.976, 1.010]* |
| Circulatory disease diagnosis ^c^ | NA  *156,953* | NA  *48,790 (31.08)* | 79,503  *79,503* | 24,723 (31.10)  *22,447 (28.23)* | *NA*  *0.872 [0.856, 0.889]* |
| Cancer diagnosis ^c^ | NA  *156,953* | NA  *20,395 (12.99)* | 79,503  *79,503* | 11,157 (14.03)  *9,948 (12.51)* | NA  *0.958 [0.933, 0.985]* |
| Education - None of the below | 154,165 | 17,971 (11.66) | 79,503 | 5,931 (7.46) | *baseline* |
| College or University degree |  | 56,296 (36.51) |  | 35,871 (45.12) | 1.346 [1.320, 1.371] |
| A levels/AS levels or equivalent |  | 44,964 (29.17) |  | 28,748 (36.16) | 1.186 [1.160, 1.212] |
| O levels/GCSEs or equivalent |  | 75,925 (49.25) |  | 42,622 (53.61) | 1.078 [1.057, 1.099] |
| CSEs or equivalent |  | 21,326 **(**13.83**)** |  | 9,978 (12.55) | 0.944 [0.920, 0.969] |
| NVQ or HND or HNC or equivalent |  | 29,203 **(**18.94**)** |  | 14,603 (18.37) | 1.062 [1.038, 1.087] |
| Other professional qualifications (e.g. nursing, teaching) |  | 47,273 **(**30.66**)** |  | 27,986 (35.20) | 1.099 [1.078, 1.120] |
| Townsend deprivation index | 156,663 | -1.60 (2.91) | 79,503 | -1.71 (2.82) | 0.986 [0.983, 0.989] |
| Death occurred ^d^ | NA  *155,168* | NA  *3,967 (2.56)* | 79,503  *79,272* | 1,615 (2.03)  *1,384 (1.75)* | NA  *0.677 [0.637, 0.720]* |
| Season – winter (December-February) | 24,181 | 4,517 (18.68) | 79,503 | 17,372 (21.85) | *NA* |
| Season – autumn (September-November) |  | 7,533 (31.15) |  | 23,243 (29.24) | 0.802 [0.769, 0.837] |
| Season – spring (March-May) |  | 5,892 (24.37) |  | 17,697 (22,26) | 0.781 [0.747, 0.816] |
| Season – summer (June-August) |  | 6,239 (25.80) |  | 21,191 (26.65) | 0.883 [0.846, 0.922] |

SD: standard deviation; CI: confidence interval; BMI: body mass index.

^a^ Mean (SD) for continuous and percentage for binary variables.

^b^ Odds ratio for participants included in our sample versus participants who were invited to wear an accelerometer but are not in our sample (reference group), for a one-unit increase in continuous variable (using variable units as described in column 1), or comparison group (indicated in column 1) versus baseline group for binary variables, or a one-category increase for ordinal categorical variables. For example, an odds ratio of 0.998 [95% CI: 0.997, 0.999] for age means that on average a UK Biobank participant is 0.2% (95% CI: 0.1%, 0.3%) less likely to be in our sample rather than having been invited but being in our sample, for each 1-year higher age.

^c^ To enable comparison between disease incidence of participants who were Invited to wear accelerometer but not in our sample versus those who have accelerometer data and are in our sample, blue italicised results show statistics for incidence defined as diagnosis prior to 1^st^ June 2013, the first date of accelerometer wear across our sample (rather than the date of accelerometer wear of each participant used in our analyses).

^d^ To enable comparison between deaths of participants who were invited to wear accelerometer but not in our sample versus those who have accelerometer data and are in our sample, the blue italicised results excluded participants (both those in our sample and those invited but not in our sample) who died before the end of the UK Biobank accelerometer data collection, set at 30^th^ December 2015 as this was the latest date a participant ended their accelerometer wear time (<http://biobank.ctsu.ox.ac.uk/showcase/field.cgi?id=90011>).
