# Supplementary material for: Association of physical activity intensity and bout length with mortality: An observational study of 79,503 UK Biobank participants
Source: PLoS Med. 2021 Sep 15;18(9):e1003757. doi: 10.1371/journal.pmed.1003757 (PMC8480840; doi:10.1371/journal.pmed.1003757)
Supplement: S2 Table — (DOCX) [file pmed.1003757.s016.docx]

## S2 Table. Summary of time spent in activity classifications on average per day

|  | **Average time per day in physical activity classes (minutes), median [IQR]** | | | |
| --- | --- | --- | --- | --- |
|  | **Complete days** | | **Other day imputed** | |
| **Main analysis (Hybrid approach)** | | | | |
|  | Median [IQR] | Number of participants with non-zero values | Median [IQR] | Number of participants with non-zero values |
| Sleep | 487.50 [448.29, 527.50] | 79,503 | 491.71 [453.00, 531.00] | 82,277 |
| Sedentary (all bout lengths) | 639.60 [562.33, 715.60] | 79,503 | 638.00 [562.71, 712.57] | 82,277 |
| Sedentary 1-15 minute bouts | 63.60 [49.25, 79.80] | 79,498 | 63.57 [49.71, 79.14] | 82,276 |
| Sedentary 16-40 bouts | 148.71 [121.00, 177.33] | 79,501 | 148.29 [122.14, 175.43] | 82,277 |
| Sedentary 41+ minutes | 417.00 [323.00, 516.29] | 79,502 | 416.29 [324.14, 512.86] | 82,277 |
| Light | 204.83 [156.20, 260.14] | 79,500 | 203.43 [156.00, 257.14] | 82,277 |
| MVPA (all bout lengths) | 91.67 [61.00, 130.00] | 79,493 | 91.00 [60.71, 128.00] | 82,275 |
| MVPA 1-9 minute bouts | 72.25 [49.67, 98.83] | 79,493 | 71.71 [49.57, 97.57] | 82,275 |
| MVPA 10-15 minute bouts | 7.50 [2.60, 14.60] | 66,484 | 7.57 [3.14, 14.29] | 72,175 |
| MVPA 16-40 minute bouts | 5.50 [0.00, 15.00] | 51,739 | 5.57 [0.00, 14.57] | 57,583 |
| MVPA 41+ minute bouts | 0.00 [0.00, 0.00] | 13,206 | 0.00 [0.00, 0.00] | 15,509 |
| **Sensitivity analysis (ML-only)** | | | | |
| Sleep | 487.60 [448.33, 527.67] | 79,503 | 491.86 [453.00, 531.14] | 82,277 |
| Sedentary (all bout lengths) | 647.57 [569.73, 723.75] | 79,503 | 645.71 [570.14, 720.71] | 82,277 |
| Sedentary 1-15 minute bouts | 40.60 [30.50, 51.67] | 79,483 | 40.71 [31.00, 51.29] | 82,,273 |
| Sedentary 16-40 minute bouts | 116.50 [92.17, 141.86] | 79,493 | 116.29 [93.57, 140.26] | 82,276 |
| Sedentary 41+ minute bouts | 484.17 [387.43, 583.17] | 79,503 | 483.29 [388.86 579.86] | 82,277 |
| Walk | 211.57 [156.67, 275.25] | 79,498 | 210.00 [156.29, 272.29] | 82,276 |
| Light | 28.71 [14.83, 49.00] | 79,060 | 28.86 [15.17, 48.29] | 82,099 |
| MVPA (all bout lengths) | 35.71 [17.40, 65.00] | 78,851 | 35.57 [17.71, 64.00] | 81,914 |
| MVPA 1-9 minute bouts | 10.86 [6.33, 16.71] | 78,323 | 10.86 [6.43, 16.32] | 81,661 |
| MVPA 10-15 minute bouts | 7.50 [3.25, 13.80] | 69,229 | 7.43 [3.43, 13.43] | 74,857 |
| MVPA 16-40 bouts | 11.40 [3.57, 25.00] | 64,019 | 11.43 [3.71, 24.29] | 69,797 |
| MVPA 41+ minutes | 0.00 [0.00, 10.50] | 28,632 | 0.00 [0.00, 10.00] | 33,428 |

Median [IQR] average time per day (minutes) spent in activity classification.

IQR: interquartile range. N (complete days sample) = 87,943; N (other day imputed sample) = 91,075.
