# Supplementary material for: Association of physical activity intensity and bout length with mortality: An observational study of 79,503 UK Biobank participants
Source: PLoS Med. 2021 Sep 15;18(9):e1003757. doi: 10.1371/journal.pmed.1003757 (PMC8480840; doi:10.1371/journal.pmed.1003757)
Supplement: S3 Table — (DOCX) [file pmed.1003757.s017.docx]

## S3 Table. Correlations between activity summary variables of main analysis (hybrid approach) and ML-only sensitivity approach

|  |  | Main summary variables (hybrid approach) | | | | | | | | | | | |  |  |  |  |  |  |  |  |  |  |
| --- | --- | --- | --- | --- | --- | --- | --- | --- | --- | --- | --- | --- | --- | --- | --- | --- | --- | --- | --- | --- | --- | --- | --- |
|  |  | Sleep | Sed | Sed 1-15 mins | Sed 16-40 mins | Sed 41+ mins | Light | MVPA | MVPA 1-9 min | MVPA 10-15 mins | MVPA 16-40 mins | MVPA 41+ mins | Sleep | Sed | Sed 1-15 mins | Sed 16-40 mins | Sed 41+ mins | Walk | Light | MVPA | MVPA 1-9 min | MVPA 10-15 mins | MVPA 16-40 mins |
| Main summary variables (hybrid approach) | Sleep | 1 |  |  |  |  |  |  |  |  |  |  |  |  |  |  |  |  |  |  |  |  |  |
|  | Sedentary | -0.437 | 1 |  |  |  |  |  |  |  |  |  |  |  |  |  |  |  |  |  |  |  |  |
|  | Sed 1-15 mins | -0.114 | -0.381 | 1 |  |  |  |  |  |  |  |  |  |  |  |  |  |  |  |  |  |  |  |
|  | Sed 16-40 mins | -0.134 | -0.128 | 0.468 | 1 |  |  |  |  |  |  |  |  |  |  |  |  |  |  |  |  |  |  |
|  | Sed 41+ mins | -0.299 | 0.920 | -0.618 | -0.482 | 1 |  |  |  |  |  |  |  |  |  |  |  |  |  |  |  |  |  |
|  | Light | -0.149 | -0.709 | 0.343 | 0.154 | -0.683 | 1 |  |  |  |  |  |  |  |  |  |  |  |  |  |  |  |  |
|  | MVPA | -0.108 | -0.586 | 0.473 | 0.224 | -0.625 | 0.279 | 1 |  |  |  |  |  |  |  |  |  |  |  |  |  |  |  |
|  | MVPA 1-9 min | -0.125 | -0.613 | 0.570 | 0.293 | -0.684 | 0.396 | 0.912 | 1.000 |  |  |  |  |  |  |  |  |  |  |  |  |  |  |
|  | MVPA 10-15 mins | -0.059 | -0.385 | 0.247 | 0.099 | -0.386 | 0.087 | 0.779 | 0.610 | 1.000 |  |  |  |  |  |  |  |  |  |  |  |  |  |
|  | MVPA 16-40 mins | -0.034 | -0.295 | 0.129 | 0.030 | -0.272 | 0.001 | 0.679 | 0.382 | 0.573 | 1 |  |  |  |  |  |  |  |  |  |  |  |  |
|  | MVPA 41+ mins | -0.022 | -0.130 | 0.028 | -0.015 | -0.106 | -0.046 | 0.373 | 0.116 | 0.205 | 0.358 | 1 |  |  |  |  |  |  |  |  |  |  |  |
| Sensitivity summary variables (ML-only) | sleep | 1.000 | -0.437 | -0.113 | -0.134 | -0.299 | -0.149 | -0.107 | -0.124 | -0.058 | -0.033 | -0.020 | 1 |  |  |  |  |  |  |  |  |  |  |
|  | sed | -0.441 | 0.999 | -0.354 | -0.109 | 0.909 | -0.717 | -0.566 | -0.591 | -0.372 | -0.286 | -0.125 | -0.442 | 1 |  |  |  |  |  |  |  |  |  |
|  | Sed 1-15 mins | -0.059 | -0.522 | 0.725 | 0.266 | -0.628 | 0.624 | 0.304 | 0.390 | 0.144 | 0.050 | -0.014 | -0.059 | -0.522 | 1 |  |  |  |  |  |  |  |  |
|  | Sed 16-40 mins | -0.077 | -0.317 | 0.407 | 0.755 | -0.553 | 0.423 | 0.172 | 0.241 | 0.067 | 0.003 | -0.034 | -0.077 | -0.316 | 0.446 | 1 |  |  |  |  |  |  |  |
|  | Sed 41+ mins | -0.334 | 0.956 | -0.475 | -0.314 | 0.955 | -0.765 | -0.540 | -0.589 | -0.337 | -0.240 | -0.092 | -0.334 | 0.956 | -0.652 | -0.566 | 1 |  |  |  |  |  |  |
|  | Walk | -0.113 | -0.572 | 0.327 | 0.163 | -0.571 | 0.630 | 0.470 | 0.498 | 0.304 | 0.232 | 0.094 | -0.112 | -0.574 | 0.490 | 0.328 | -0.608 | 1 |  |  |  |  |  |
|  | Light | -0.102 | -0.316 | 0.141 | 0.053 | -0.298 | 0.440 | 0.177 | 0.209 | 0.104 | 0.049 | 0.013 | -0.101 | -0.313 | 0.158 | 0.104 | -0.300 | 0.091 | 1 |  |  |  |  |
|  | MVPA | -0.090 | -0.329 | 0.210 | 0.058 | -0.321 | 0.262 | 0.446 | 0.428 | 0.314 | 0.266 | 0.172 | -0.090 | -0.319 | 0.125 | 0.057 | -0.289 | -0.001 | 0.108 | 1 |  |  |  |
|  | MVPA 1-9 min | -0.095 | -0.327 | 0.297 | 0.145 | -0.360 | 0.298 | 0.396 | 0.444 | 0.251 | 0.153 | 0.049 | -0.095 | -0.316 | 0.209 | 0.144 | -0.318 | 0.092 | 0.133 | 0.710 | 1 |  |  |
|  | MVPA 10-15 mins | -0.081 | -0.262 | 0.218 | 0.093 | -0.278 | 0.231 | 0.335 | 0.359 | 0.233 | 0.151 | 0.051 | -0.080 | -0.253 | 0.135 | 0.087 | -0.244 | 0.007 | 0.120 | 0.746 | 0.649 | 1 |  |
|  | MVPA 16-40 mins | -0.076 | -0.284 | 0.181 | 0.044 | -0.276 | 0.235 | 0.372 | 0.369 | 0.267 | 0.220 | 0.092 | -0.076 | -0.276 | 0.107 | 0.046 | -0.249 | -0.014 | 0.106 | 0.885 | 0.599 | 0.632 | 1 |
|  | MVPA 41+ mins | -0.050 | -0.207 | 0.070 | -0.017 | -0.176 | 0.128 | 0.326 | 0.254 | 0.238 | 0.256 | 0.264 | -0.050 | -0.202 | 0.026 | -0.019 | -0.163 | -0.030 | 0.029 | 0.756 | 0.293 | 0.333 | 0.468 |

Yellow highlighted cells are those comparing the same activity variable generated using the main (100mg threshold) and sensitivity (prediction only) approaches.

Green highlighted cells are those comparing the overall time spent sedentary or in MVPA, with time spent in bout length categories.
