# Supplementary material for: Association of physical activity intensity and bout length with mortality: An observational study of 79,503 UK Biobank participants
Source: PLoS Med. 2021 Sep 15;18(9):e1003757. doi: 10.1371/journal.pmed.1003757 (PMC8480840; doi:10.1371/journal.pmed.1003757)
Supplement: S4 Table — (DOCX) [file pmed.1003757.s018.docx]

## S4 Table. Associations of transferring time between overall activity categories, with all-cause mortality

a) Main analysis (hybrid approach)

|  |  | **Association of transferring 10 minutes from baseline to comparison activity category, on average per day** | | | | | | | | | | | |
| --- | --- | --- | --- | --- | --- | --- | --- | --- | --- | --- | --- | --- | --- |
|  |  | **Completed days version** | | | | | | **Other day imputed version** | | | | | |
|  |  | **Unadjusted** | | **Adjusted ^a^** | | **Sensitivity ^b^** | | **Unadjusted** | | **Adjusted ^a^** | | **Sensitivity ^b^** | |
| **Baseline** | **Comparison** | **Estimate** | **Schoenfeld residual P value** | **Estimate** | **Schoenfeld residual P value** | **Estimate** | **Schoenfeld residual P value** | **Estimate** | **Schoenfeld residual P value** | **Estimate** | **Schoenfeld residual P value ^b^** | **Estimate** | **Schoenfeld residual P value** |
| Sleep | All other | 1.007 [1.000, 1.014] | 0.334 | 1.001 [0.994, 1.007] | 0.149 | 1.003 [0.996, 1.010] | 0.160 | 1.004 [0.997, 1.011] | 0.101 | 0.998 [0.992, 1.005] | 0.045 | 1.000 [0.993, 1.007] | 0.046 |
|  | Sedentary | 1.014 [1.007; 1.020] | 0.012 | 1.008 [1.001, 1.014] | 0.039 | 1.009 [1.003, 1.016] | 0.011 | 1.012 [1.005, 1.018] | 0.067 | 1.006 [0.999, 1.012] | 0.160 | 1.007 [1.001, 1.013] | 0.061 |
|  | Light | 0.985 [0.976, 0.994] | 0.038 | 0.991 [0.982, 1.000] | 0.229 | 0.991 [0.982, 1.000] | 0.091 | 0.982 [0.973, 0.991] | 0.272 | 0.988 [0.980, 0.997] | 0.778 | 0.988 [0.979, 0.997] | 0.446 |
|  | MVPA | 0.945 [0.931, 0.958] | 0.064 | 0.949 [0.936, 0.963] | 0.118 | 0.941 [0.928, 0.954] | 0.048 | 0.939 [0.926, 0.952] | 0.53 | 0.943 [0.930, 0.957] | 0.667 | 0.935 [0.922, 0.948] | 0.411 |
| Sedentary | All other | 0.968 [0.965, 0.972] | 0.061 | 0.978 [0.974, 0.983] | 0.149 | 0.974 [0.970, 0.978] | 0.064 | 0.968 [0.964, 0.972] | 0.248 | 0.978 [0.974, 0.982] | 0.470 | 0.974 [0.970, 0.978] | 0.261 |
|  | Light | 0.971 [0.965, 0.978] | 0.038 | 0.984 [0.976, 0.991] | 0.229 | 0.982 [0.975, 0.989] | 0.091 | 0.970 [0.963, 0.977] | 0.272 | 0.983 [0.976, 0.990] | 0.778 | 0.981 [0.974, 0.988] | 0.446 |
|  | MVPA | 0.932 [0.920, 0.943] | 0.064 | 0.942 [0.930, 0.954] | 0.118 | 0.932 [0.921, 0.944] | 0.048 | 0.928 [0.916, 0.939] | 0.53 | 0.938 [0.926, 0.950] | 0.667 | 0.928 [0.917, 0.940] | 0.411 |
| Light | All other | 1.043 [1.036, 1.050] | 0.053 | 1.028 [1.020, 1.035] | 0.330 | 1.033 [1.026, 1.040] | 0.139 | 1.046 [1.039, 1.053] | 0.351 | 1.030 [1.022, 1.037] | 0.980 | 1.035 [1.028, 1.043] | 0.603 |
|  | MVPA | 0.959 [0.944, 0.975] | 0.064 | 0.958 [0.942, 0.974] | 0.118 | 0.950 [0.934, 0.966] | 0.048 | 0.956 [0.941, 0.972] | 0.53 | 0.954 [0.939, 0.971] | 0.667 | 0.946 [0.930, 0.962] | 0.411 |
| MVPA | All other | 1.091 [1.078, 1.105] | 0.027 | 1.071 [1.058, 1.084] | 0.080 | 1.084 [1.071, 1.097] | 0.028 | 1.097 [1.084, 1.111] | 0.317 | 1.076 [1.063, 1.089] | 0.529 | 1.090 [1.077, 1.103] | 0.297 |

b) Sensitivity analysis using ML-only approach

|  |  | **Association of transferring 10 minutes from baseline to comparison activity category, on average per day** | | | | | | | | | | | |
| --- | --- | --- | --- | --- | --- | --- | --- | --- | --- | --- | --- | --- | --- |
|  |  | **Completed days version** | | | | | | **Other day imputed version** | | | | | |
|  |  | **Unadjusted** | | **Adjusted ^a^** | | **Sensitivity ^b^** | | **Unadjusted** | | **Adjusted ^a^** | | **Sensitivity ^b^** | |
| **Baseline** | **Comparison** | **Estimate** | **Schoenfeld residual P value ^c^** | **Estimate** | **Schoenfeld residual P value ^c^** | **Estimate** | **Schoenfeld residual P value ^c^** | **Estimate** | **Schoenfeld residual P value ^c^** | **Estimate** | **Schoenfeld residual P value ^c^** | **Estimate** | **Schoenfeld residual P value ^c^** |
| Sleep | All other | 1.007 [1.000, 1.014] | 0.334 | 1.001 [0.994, 1.007] | 0.149 | 1.003 [0.996, 1.010] | 0.160 | 1.004 [0.997, 1.012] | 0.102 | 0.998 [0.992, 1.005] | 0.045 | 1.000 [0.993, 1.007] | 0.046 |
|  | Sedentary | 1.013 [1.007, 1.020] | 0.014s | 1.007 [1.001, 1.014] | 0.049 | 1.009 [1.003, 1.016] | 0.016 | 1.011 [1.005, 1.018] | 0.076 | 1.005 [0.999, 1.011] | 0.189 | 1.007 [1.001, 1.014] | 0.084 |
|  | Walk | 0.968 [0.959, 0.976] | 0.129 | 0.974 [0.965, 0.982] | 0.379 | 0.971 [0.963, 0.979] | 0.18 | 0.963 [0.955, 0.971] | 0.77 | 0.969 [0.961, 0.977] | 0.795 | 0.966 [0.958, 0.975] | 0.849 |
|  | Light | 1.011 [0.993, 1.029] | 0.244 | 1.012 [0.995, 1.030] | 0.345 | 1.011 [0.993, 1.029] | 0.246 | 1.011 [0.994, 1.029] | 0.589 | 1.013 [0.996, 1.031] | 0.751 | 1.011 [0.994, 1.029] | 0.592 |
|  | MVPA | 0.961 [0.947, 0.976] | 0.054 | 0.972 [0.957, 0.987] | 0.195 | 0.966 [0.952, 0.981] | 0.136 | 0.957 [0.942, 0.972] | 0.095 | 0.968 [0.953, 0.983] | 0.294 | 0.962 [0.948, 0.977] | 0.226 |
| Sedentary | All other | 0.969 [0.965, 0.973] | 0.044 | 0.979 [0.975, 0.983] | 0.119 | 0.975 [0.971, 0.979] | 0.049 | 0.968 [0.964, 0.972] | 0.188 | 0.979 [0.974, 0.983] | 0.392 | 0.974 [0.970, 0.978] | 0.207 |
|  | Walk | 0.955 [0.949, 0.961] | 0.129 | 0.967 [0.961, 0.973] | 0.379 | 0.962 [0.956, 0.968] | 0.18 | 0.952 [0.946, 0.958] | 0.77 | 0.964 [0.958, 0.970] | 0.795 | 0.959 [0.953, 0.965] | 0.849 |
|  | Light | 0.997 [0.981, 1.014] | 0.244 | 1.005 [0.988, 1.022] | 0.345 | 1.002 [0.985, 1.019] | 0.246 | 1.000 [0.983, 1.017] | 0.589 | 1.008 [0.991, 1.026] | 0.751 | 1.004 [0.987, 1.022] | 0.592 |
|  | MVPA | 0.949 [0.936, 0.962] | 0.054 | 0.965 [0.951, 0.979] | 0.195 | 0.957 [0.944, 0.971] | 0.136 | 0.946 [0.933, 0.960] | 0.095 | 0.963 [0.949, 0.977] | 0.294 | 0.956 [0.942, 0.970] | 0.226 |
| Walk | All other | 1.047 [1.040, 1.053] | 0.118 | 1.033 [1.027, 1.040] | 0.369 | 1.039 [1.033, 1.046] | 0.168 | 1.050 [1.044, 1.057] | 0.734 | 1.036 [1.030, 1.043] | 0.806 | 1.042 [1.036, 1.049] | 0.821 |
|  | Light | 1.044 [1.025, 1.064] | 0.244 | 1.040 [1.021, 1.059] | 0.345 | 1.041 [1.022, 1.061] | 0.246 | 1.050 [1.031, 1.070] | 0.589 | 1.046 [1.026, 1.065] | 0.751 | 1.047 [1.027, 1.067] | 0.592 |
|  | MVPA | 0.993 [0.978, 1.009] | 0.054 | 0.998 [0.983, 1.014] | 0.195 | 0.995 [0.980, 1.011] | 0.136 | 0.994 [0.978, 1.009] | 0.095 | 0.999 [0.983, 1.015] | 0.294 | 0.996 [0.980, 1.012] | 0.226 |
| Light | All other | 1.021 [1.004, 1.038] | 0.580 | 1.004 [0.987, 1.022] | 0.614 | 1.013 [0.996, 1.030] | 0.517 | 1.021 [1.004, 1.039] | 0.918 | 1.004 [0.987, 1.021] | 0.879 | 1.013 [0.995, 1.030] | 0.985 |
|  | MVPA | 0.951 [0.930, 0.973] | 0.054 | 0.960 [0.938, 0.982] | 0.195 | 0.956 [0.934, 0.978] | 0.136 | 0.946 [0.924, 0.968] | 0.095 | 0.955 [0.933, 0.978] | 0.294 | 0.952 [0.930, 0.974] | 0.226 |
| MVPA | All other | 1.055 [1.040, 1.070] | 0.044 | 1.035 [1.020, 1.050] | 0.196 | 1.046 [1.031, 1.061] | 0.147 | 1.058 [1.043, 1.073] | 0.075 | 1.037 [1.022, 1.052] | 0.289 | 1.049 [1.033, 1.064] | 0.236 |

Hazard ratio of spending 10 minutes less time per day in baseline category coupled with spending 10 minutes more time per day in comparison category.

**^a^** Adjusted model covariates: age at accelerometer wear, sex, ethnicity, season, smoking, SEP (education, Townsend deprivation index, income), BMI, and three indicators denoting whether the participant had cardiovascular disease, cancer or respiratory disease prior to accelerometer wear.

**^b^** Sensitivity: model a, without adjustment for BMI, and cardiovascular disease, cancer or respiratory disease.

**^c^** Schoenfeld residual P value, is the P value of the correlation between log transformed survival time and the scaled Schoenfeld residuals (cox.zph function in R survival package).

## 
