# Supplementary material for: Association of physical activity intensity and bout length with mortality: An observational study of 79,503 UK Biobank participants
Source: PLoS Med. 2021 Sep 15;18(9):e1003757. doi: 10.1371/journal.pmed.1003757 (PMC8480840; doi:10.1371/journal.pmed.1003757)
Supplement: S1 Text — Section A: Description of participant flow. Section B: Potential confounders. Section C: Missing data assumptions and imputation of accelerometer data. Section D: Deriving activity summary variables. Section E: Estimating the association of less time in a given activity category, when coupled with more time in another category. Section F: Estimating the association of less time in a given MVPA bout length stratum, when coupled with more time in another activity category/MVPA bout length stratum. Section G: Possible bias due to conditioning on a collider. MVPA, moderate-vigorous physical activity. (DOCX) [file pmed.1003757.s021.docx]

Association of physical activity intensity and bout length with mortality: an observational study of 79,503 UK Biobank participants

LAC Millard et al.

[S1 TEXT. 2](#_Toc80104162)

[Section A. Description of participant flow 2](#_Toc80104163)

[Section B. Potential confounders 2](#_Toc80104164)

[Section C. Missing data assumptions and imputation of accelerometer data 2](#_Toc80104165)

[Section D. Deriving activity summary variables 3](#_Toc80104166)

[Section E. Estimating the association of less time in a given activity category, when coupled with more time in another category 4](#_Toc80104167)

[Section F. Estimating the association of less time in a given MVPA bout length stratum, when coupled with more time in another activity category / MVPA bout length stratum 5](#_Toc80104168)

[Section G. Possible bias due to conditioning on a collider 5](#_Toc80104169)

[References 6](#_Toc80104170)

# S1 TEXT.

## Section A. Description of participant flow

Of the 103,684 participants with accelerometer data we removed 4695 participants with an accelerometer data problem (e.g. an unexpectedly large data size), 8 participants whose accelerometer data could not be calibrate, and 4467 participants with less than 72 hours of accelerometer wear time (UK Biobank participants were asked to wear the accelerometer continuously for 7 days). Following these exclusions, we removed a further 10,338 participants with missing data for one or more potential confounding factors (ethnicity, education, Townsend deprivation index, household income, smoking status, and number of cancer and non-cancer illnesses). This resulted in a sample size of 84,176 participants, although a slightly smaller sample was used in analyses after dealing with missing periods in the accelerometer data (see Results section of main paper).

## Section B. Potential confounders

Education level was self-reported during the touchscreen questionnaire and grouped into 7 categories: “College or University degree”, “A levels/AS levels or equivalent” (school exams usually taken age 17-18 years in the UK), “O levels/GCSEs or equivalent” (school exams usually taken age 15-16 years in the UK), “CSEs or equivalent” (school exams usually taken age 16 years in the UK that are a lower academic level than O levels/GCSEs), “NVQ or HND or HNC or equivalent” (Vocational, rather than academic qualifications taken usually between age 16-19 years in the UK), “Other (non-university) professional qualifications e.g.: nursing, teaching” and “None of the above” (the baseline category in our analyses). Household total income (<£18,000, £18,000-£30,999, £31,000-£51,999, £52,000-£100,000, >£100,000) was obtained from self-report. Townsend deprivation index was calculated immediately prior to participants joining UK Biobank using their self-reported postcode of residence. We grouped ethnic origin into the following four categories: white; black or black British; Asian or Asian British; or other.

## Section C. Missing data assumptions and imputation of accelerometer data

Missing periods may be systematically different to the non-missing periods. For example, if a participant chose to remove their device while swimming, their activity levels (both overall and within moderate activity bouts) will be underestimated. In general, if this is the case then associations of activity levels with a potential outcome or risk factor may be biased. We note that bias in the activity variable (e.g. systematically lower values than the true value among swimmers) would not necessarily cause bias in subsequent analyses using this variable, as this depends on the particular analysis performed. It may be the case that missing periods in the accelerometer data are not systematically different to non-missing periods, i.e. the accelerometer data is missing completely at random. For example, some technological failures of accelerometers may be due to chance.

We used of two approaches to attempt to deal with missing data – a complete days approach that includes only whole days with no missing data, and an ‘other day’ imputed approach, that fills in a participants missing periods with non-missing periods from their other days. If the days of accelerometer data are missing completely at random (MCAR_days_) such that there are no systematic differences between the days with and without missing accelerometer data, then activity summary variables derived from complete days data will be unbiased, hence this missingness will not bias results of subsequent analyses [1]. The MCAR_days_ assumption of the complete days approach may be violated. For example, if participants are more likely to remove the device during physical activity then the days with missing data will, on average, have higher activity levels compared with days with no missing data. However, even when MCAR_days_ does not hold, analyses using complete days data may still be unbiased depending on the specific further analysis in which they are used [1]. For our particular analyses, assessing the association of time spent in activity bouts with all-cause mortality, if a third factor (e.g. participating in swimming) affects all-cause mortality and our activity bout exposure variable via non-wear, then our associations may be biased.

In general, imputation may help to reduce the amount of excluded data and relax the missing data assumptions, such that missing at random (MAR) (or sometimes missing not at random [MNAR]) may be assumed rather than MCAR [1]. We used ‘other day imputation’ to impute missing periods. This involved finding all other periods of accelerometer data on other days, that are during the same time period and have no missing data. One of these periods is then randomly chosen as the imputed sequence for the missing region. In general, other day imputation may help to reduce bias in the derived activity variables and hence bias in subsequent analyses that use these statistics. Under the assumption that regions at the same time as the missing period, but on other days, are representative of that particular missing period, then the derived activity statistics may be less biased. It may however be more likely that missing regions are systematically different to the same time period on other days. For example, if a person takes their device off to swim and does not swim on the same time each day, then the activity in the missing periods (when they are swimming) will be systematically different to the activity in the non-missing periods at the same time period on other days (when they are not swimming). In this case ‘other day’ imputation may still help to reduce bias in the derived activity variables. This is because, if days with missing data are systematically different to days without missing data (other than the missing region itself) then other day imputation will enable information from (the non-missing time periods on) these systematically different days to be incorporated into the derived activity variables. Similarly, if the accelerometer data are MCAR_day_ then the summary activity variables derived using the other day imputed data will be unbiased and more precise than the complete days version.

## Section D. Deriving activity summary variables

We used the Doherty machine learning model (part of the UK Biobank accelerometer analysis tool [https://github.com/activityMonitoring/biobankAccelerometerAnalysis/]) to classify each 1 minute epoch of accelerometer data to an activity category (using the second version of their model as used in their GWAS study) [2]. In brief, they asked 143 participants to wear an Axivity AX3 accelerometer and a GoPro camera over a 24-hour period, of which 132 participants had valid data [3]. These data were then used to create a labelled dataset, where each minute of accelerometer data was labelled with an activity from the compendium of physical activities [4]. They then used these labelled data to train a machine learning model to predict these activities. The final model grouped the activities into the set of high-level categories: sleep, sedentary, light, walk and moderate-vigorous, predominantly based on the metabolic-equivalent (MET) assignments in the compendium of physical activities. For example, ‘walking the dog’ (MET=3) was assigned into the walk category whereas ‘mowing the lawn’ (MET=5.5) was assigned to the moderate-vigorous category. The performance of their model varies across the activity classes (see Supplementary table 1 in [2] for the contingency table). For example, 81% of 1 minute sedentary epochs were correctly identified as sedentary, whereas 58% of moderate-vigorous epochs were correctly identified.

We identified, for each participant, contiguous sequences of 1 minute epochs with a given activity category; these contiguous epochs are referred to as *activity bouts* and can be of any length so long as the participant remains in the same activity category. We refer to this approach to bout generation as the *prediction-based* approach. As described above, the activity predictions for some classes are less accurate than for others. For this reason, as a sensitivity analysis, we use an alternative approach to identify activity categories, that incorporates the machine learning predictions for well-predicted categories, with an approach using an activity level threshold to identify MVPA. We refer to this approach to generating bouts as the *hybrid* approach.

Activity bouts are identified using the hybrid threshold as follows. We first converted the raw accelerometer data into 1 minute intervals called epochs, using the UK Biobank accelerometer analysis tool [5]. This process generates, for each 1-minute period (consisting of a series of raw activity measurements), a single measure representing the overall activity in the epoch, called the average vector magnitude (AVM):

$$AVM= \frac{1}{n}\sum_{i=1:n} \left( \sqrt{x_{i}^{2}+ y_{i}^{2}+z_{i}^{2}}-1 \right)$$

where *x_i_*, *y_i_* and *z_i_* are the activity levels – measured in milli-gravity (m-grav) – along the three axes, for each raw activity measurement *i* within an epoch, and one is subtracted to remove gravity. AVM is a measure of the average activity across the three axes of the accelerometer, for each epoch.

In contrast to the accelerometer data measured in ‘counts per minute’ [cite], there is currently limited evidence on the values of AVM that should be used as thresholds between sedentary, low, moderate and vigorous physical activity. One small study exists that suggests a threshold of circa 100 m-grav is appropriate to define MVPA [6]. In UK Biobank on average 93.2% of a participant’s time was spent below 100m-grav. To generate activity bouts in moderate-vigorous activity we identified, for each participant, contiguous sequences of 1 minute epochs with AVM>=100m-grav; these bouts can be of any length so long as the participant remains above the predefined threshold. All remaining 1 minute epochs that were not assigned to the moderate-vigorous category were then used to identify sleep and sedentary bouts, using the predictions as described above. Thus the sleep and sedentary bouts are the same as the prediction-based approach, except where the bout included one or more epochs with AVM>=100m-grav. The remaining periods of time not assigned to moderate-vigorous, sleep or sedentary were assigned as bouts of *light* activity.

## Section E. Estimating the association of less time in a given activity category, when coupled with more time in another category

Within 1 day there are a finite number of minutes (1440), such that as the frequency of one activity category increases, this much be coupled with a decrease in the frequency of one or more of the other activity categories. For example, reducing time spent sedentary may be accompanied by an increase in time spent sleeping, or in moderate-vigorous activity. For this reason we model associations in terms of transferring time between activity categories, in a similar way to our previous activity bigrams approach [7]. We assign, in turn, one activity category as the baseline and estimate the hazard per 10 minutes higher of another activity category, when coupled with 10 minute less of the baseline category. We include all other activity categories in the model (i.e. this model is estimated four times with either sleep, sedentary, light or moderate-vigorous as the baseline). For example, we use the following (unadjusted) model to estimate the association of transferring 1 minute of time from sedentary to all other activity categories:

$$h(t)= {h_{0}\left( t \right)\times\exp(\beta}_{sleep}\times x_{sleep}+\beta_{walk}\times x_{walk}+ \beta_{light}\times x+ \beta_{mod-vig}\times x_{mod-vig})$$

where *x_c_* is the average time spent in activity category *c* per day. Parameter $\beta_{c}$ is the log hazard ratio of transferring 1 minute of time from the sedentary activity category to activity category *c*, on average per day. We note that swapping the baseline, *b*, and comparison, *c*, activity categories results in a reciprocal model with estimate, i.e. $\beta_{b}= -\beta_{c}$. We multiply all estimated β_x_ by 10 such that hazard ratios, exp(βx) are for the transfer of 10 minutes of time between activity categories.

## Section F. Estimating the association of less time in a given MVPA bout length stratum, when coupled with more time in another activity category / MVPA bout length stratum

As described in the main paper, we estimated the hazard per 10 minutes less time spent in MVPA bouts of a given length, when coupled with 10 minutes more of another activity category or MVPA bout length stratum. For example, we use the following model to estimate the association of transferring 1 minute of time from sedentary bouts of length 1-15 minutes, to all other activity or sedentary bout categories:

v

$$h(t)= h_{0}\left( t \right)\times\exp(\beta_{sleep}\times x_{sleep}+{\beta_{sed16-40}\times x_{sed16-40}+ \beta_{sed41+}\times x_{sed41+}+\beta}_{walk}\times x_{walk}+ \beta_{light}\times x_{light}+ \beta_{mod-vig}\times x_{mod-vig})$$

Where, for example, $\beta_{sed41+}$ is the log hazard ratio of transferring 1-minute of time from sedentary bouts of length 1-15 minutes to sedentary bouts of 41+ minutes, on average per day. For example, transferring 6x10 minute sedentary bouts to one 60 minute bout is associated with a ${60\times\beta}_{set41+}$ log hazard ratio. Again, we multiply all estimated β_x_ by 10 such that hazard ratios, exp(βc) are for the transfer of 10 minutes of time between activity categories.

## Section G. Possible bias due to conditioning on a collider

Depending on the selection mechanism – i.e. how selection relates to the exposure and outcomes variables – estimates of the association of an exposure on an outcome may be biased by a particular form of collider bias ­– selection-induced collider bias [8]. In general, when testing the association of an exposure (X) on an outcome (Y), collider bias may occur when a third variable (C), or a descendent of C, is conditioned upon in analyses, opening up a non-causal pathway between X and Y [8,9]. Selection induced collider bias may occur when variable C (or a descendent of C) represents whether a person is selected into the sample (i.e. when participation in the study is conditioned upon) [8]. Hence, estimates of association between our activity phenotypes and all-cause mortality may be biased if the activity phenotype affects selection into the study (see S13 Fig for an example) [10]. Furthermore, collider bias could also be induced if a covariate thought to be a confounder is adjusted (i.e. conditioned upon) in regression models but is in reality a collider instead.

## References

1. White IR, Carlin JB. Bias and efficiency of multiple imputation compared with complete-case analysis for missing covariate values. Stat Med. 2010;29: 2920–2931. doi:10.1002/sim.3944

2. Doherty A, Smith-Byrne K, Ferreira T, Holmes M V, Holmes C, Pulit SL, et al. GWAS identifies 14 loci for device-measured physical activity and sleep duration. Nat Commun. 2018;9: 5257. doi:10.1038/s41467-018-07743-4

3. Willetts M, Hollowell S, Aslett L, Holmes C, Doherty A. Statistical machine learning of sleep and physical activity phenotypes from sensor data in 96,220 UK Biobank participants. Sci Rep. 2018;8: 7961. doi:10.1038/s41598-018-26174-1

4. Ainsworth BE, Haskell WL, Herrmann SD, Meckes N, Bassett DRJR, Tudor-Locke C, et al. 2011 Compendium of Physical Activities: A Second Update of Codes and MET Values. Med Sci Sport Exerc. 2011;43: 1575–1581.

5. Doherty A, Jackson D, Hammerla N, Plötz T, Olivier P, Granat MH, et al. Large Scale Population Assessment of Physical Activity Using Wrist Worn Accelerometers: The UK Biobank Study. PLoS One. 2017;12: e0169649. doi:10.1371/journal.pone.0169649

6. Bell JA, Hamer M, van Hees VT, Singh-Manoux A, Kivimaki M, Sabia S. Healthy obesity and objective physical activity. Am J Clin Nutr. 2015;102: 268–275. doi:10.3945/ajcn.115.110924

7. Millard LAC, Tilling K, Lawlor DA, Flach PA, Gaunt TR. Physical activity phenotyping with activity bigrams, and their association with BMI. Int J Epidemiol. 2017;46. doi:10.1093/ije/dyx093

8. Munafò MR, Tilling K, Taylor AE, Evans DM, Davey Smith G. Collider Scope: When selection bias can substantially influence observed associations. Int J Epidemiol. 2017;47: 226–235. doi:https://doi.org/10.1101/079707

9. Pearl J, Glymour M, Jewell NP. Causal inference in statistics: A primer. John Wiley & Sons; 2016.

10. Hughes RA, Davies NM, Davey Smith G, Tilling K. Selection bias when estimating average treatment effects using one-sample instrumental variable analysis. Epidemiology. 2019;30: 350–357. doi:10.1097/EDE.0000000000000972

11. Richmond RC, Davey Smith G, Ness AR, den Hoed M, McMahon G, Timpson NJ. Assessing causality in the association between child adiposity and physical activity levels: a Mendelian randomization analysis. PLoS Med. 2014;11: e1001618. doi:10.1371/journal.pmed.1001618
