# Supplementary material for: Association of physical activity intensity and bout length with mortality: An observational study of 79,503 UK Biobank participants
Source: PLoS Med. 2021 Sep 15;18(9):e1003757. doi: 10.1371/journal.pmed.1003757 (PMC8480840; doi:10.1371/journal.pmed.1003757)
Supplement: Attachment — Submitted filename: reviewer-responses20201028.docx [file pmed.1003757.s022.docx]

Requests from the editors:
Title
Please revise your title according to PLOS Medicine's style. Your title must be nondeclarative and not a question. It should begin with main concept if possible. "Effect of" should be used only if causality can be inferred, i.e., for an RCT. Please place the study design ("A randomized controlled trial," "A retrospective study," "A modelling study," etc.) in the subtitle (ie, after a colon).

We have now changed the title adding ‘prospective’ after the colon (and updating the sample size):

“Association of changing physical activity intensity and bout length with mortality: a **prospective** study of 79,503 participants in UK Biobank”.

Abstract
* Please ensure that all numbers presented in the abstract are present and identical to numbers presented in the main manuscript text.

These have been checked and they are identical.

* Please include the study design, population and setting, number of participants, years during which the study took place, length of follow up, and main outcome measures. * Please quantify the main results (with 95% CIs and p values).

We have now added the study design, setting information and the years during which the study took place, to the abstract on page 2:

“We used data on 79,503 **adult** participants from **the population-based** UK Biobank cohort, which **recruited participants between 2006 and 2010 (mean age at accelerometer wear 62.1 years [SD=7.9], 54.5% women; mean length of follow up 5.1 years [SD=0.73])**.”

We have also added P values to our abstract results.

Please rephrase “we found little evidence…” to be more specific. The same goes for the sentence in the main conclusions

We have now updated this to be more specific, changing this (on lines 101-103) from:

“We found little evidence to suggest that the impact of MVPA differs depending on whether it is obtained from several short bouts or fewer longer bouts …”

To:

“**Our findings** suggest that the impact of MVPA **does not** differ depending on whether it is obtained from several short bouts or fewer longer bouts …”

Limitations of this observational study must be stated more explicitly and please provide 2-3 limitations
“supporting recent policy changes in some countries” is a bit vague and we suggest removing this or rephrasing to be more specific.

We have now included two further limitations on lines 27-96:

“Key limitations of our study are potential residual confounding, **the limited length of follow up, and use of a select sample of the UK population (UK Biobank had a 5% recruitment response).**”

We have now improved the detail for “supporting recent policy changes in some countries” (lines 103/105):

“…supporting the recent **removal of the requirement that MVPA should be accumulated in bouts of 10 minutes or more from UK and US policy.**”

Author Summary
At this stage, we ask that you include a short, non-technical Author Summary of your research to make findings accessible to a wide audience that includes both scientists and non-scientists. The Author Summary should immediately follow the Abstract in your revised manuscript. This text is subject to editorial change and should be distinct from the scientific abstract. Please see our author guidelines for more information: <https://journals.plos.org/plosmedicine/s/revising-your-manuscript#loc-author-summary>

We have included an author summary on page 4 of the manuscript.

Throughout: please use square brackets for references and these should be placed after all punctuation. Please format the bibliography using Vancouver style

We have now updated the references and bibliography.

Introduction
I would suggest removing this sentence as it appears to be based on conjecture “All those studies grouped bouts ≥10 mins together, probably because they lacked power to explore more bouts length categories”

Thank you, we have removed the latter part of this sentence (line 181-189) so it now says “They all grouped bouts $\geq$10 mins together.”

Suggest rephrasing this “have used two statistics…”

We were unclear why the editor is pointing out this part of the sentence. We assumed it is our use of ‘statistics’ so have now amended this (on line 189) from:

“Other studies have used two statistics to characterise MVPA bouts …”

To:

“Other studies have used two **summary variables** to characterise MVPA bouts …”

We are happy to revise further if this is not the issue and the editor could provide more details.

“Our study is the largest observational study of activity bouts to date and is the first to assess associations of MVPA” please add “to our knowledge” to temper assertions of primacy

Thank you, we have now added ‘to our knowledge’ to this sentence (line 202), and also updated this sentence to describe our study as the second, rather than the 1^st^ to assess associations of MVPA bout length with all-cause mortality.

Methods

The link in “Analyses were performed in R version 3.5.1, Matlab r2015a or Stata version 15, and all of our analysis code is available at [https://github.com/MRCIEU/UKBActivityBoutLength” is not working, please provide a working link here and in the discussion. This link should also be provided in the data availability statement.

The URLs were correct in the previous version, but not hyperlinked. We have now added hyperlinks so that the reader can click to open.

Here is the URL for the GitHub repository: <https://github.com/MRCIEU/UKBActivityBoutLength/>.

Results
Please provide p values along with 95% CI as needed. Please note all p-values should be exact, unless p<0.001

We have now added P values to accompany 95% confidence intervals in the paper. For example, on lines 520 and 523.

Discussion
Please temper associations of primacy by adding “to our knowledge” for example in Line 309

We have added ‘to our knowledge’ to this sentence (now on lines 675-677), and we now also cite one previous study also:

“To our knowledge, there is only one existing study that assessed the association of MVPA bout length with mortality; it was considerably smaller than our study (N=4,840) and consistent with our findings, found no strong evidence of association between MVPA bout length and mortality [8].”

Please ensure that the study is reported according to the STROBE guideline, and include the completed STROBE  checklist as Supporting Information. When completing the checklist, please use section and paragraph numbers, rather than page numbers. Please add the following statement, or similar, to the Methods: "This study is reported as per the Strengthening the Reporting of Observational Studies in Epidemiology (STROBE) guideline (S1 Checklist)."

We now include the STROBE checklist as supplementary material, and added the above sentence to the Methods section on lines 229/230.

Did your study have a prospective protocol or analysis plan? Please state this (either way) early in the Methods section.

We have added the following text on lines 209-230:

“The analysis plan was developed by LACM, DAL, KT and TRG prior to analyses beginning. We initially sought to investigate the impact of physical activity bout length on body mass index, but changed this to all-cause mortality because body mass index is measured prior to accelerometer wear in UK Biobank. Following reviewers’ comments we made one change to our main analysis: splitting our MVPA 1-15 minute bout length stratum into two categories: 1-9 minutes and 10-15 minutes, so that the different impact of <10 and >=10 minute bouts could be directly assessed. We also added two additional sensitivity analysis, 1) using the isometric log ratio transformation as an alternative approach to analysing compositional data, and 2) repeating our main analyses excluding the first year and first two years of follow-up to explore whether undiagnosed prevalent disease might confound our results.”

a) If a prospective analysis plan (from your funding proposal, IRB or other ethics committee submission, study protocol, or other planning document written before analyzing the data) was used in designing the study, please include the relevant prospectively written document with your revised manuscript as a Supporting Information file to be published alongside your study, and cite it in the Methods section. A legend for this file should be included at the end of your manuscript.

Not applicable, we do not have a formal plan to include but instead describe the analytical changes made during the project, as detailed above.

b) If no such document exists, please make sure that the Methods section transparently describes when analyses were planned, and when/why any data-driven changes to analyses took place.

Please see response above.

c) In either case, changes in the analysis-- including those made in response to peer review comments-- should be identified as such in the Methods section of the paper, with rationale.

Please see response above.

**Reviewer #1: To the Authors**

You have examined the role of substituting time spent asleep, sedentary, in light and moderate-to-vigorous intensity activity (MVPA) on the associations with mortality, including examining the role of bout length of MVPA (1-15min, 16-40min, and 41+ min bouts). The substitution of time across intensities has been addressed in recent meta-analyses with more deaths and is less novel but the influence of bout length is a matter of much current debate, since some health authorities have recently changed their public health recommendations for MVPA to occur in at least 10-min bouts; it therefore seems rather odd that this paper has defined short bout durations across this divide of 10-min - this is where the uncertainty still lies and the current analysis is a lost opportunity for addressing this issue; it is vital to re-parameterise the bouts to explore the <10-min bout duration range and then compare that to the 10-min and above estimates. The standard time format of derived activity measures in UK Biobank is 5-sec bouts so there is ample opportunity to address this issue.

We appreciate that this is where there is a policy debate around <10 minutes versus 10 minutes + and two reviewers have suggested strata that split on this duration. We have therefore amended our bout length strata, splitting our MVPA 1-15 minute stratum into two categories: 1-9 minutes and 10-15 minutes.

We have updated this throughout the paper, e.g. in the methods on lines 354-356:

“For MVPA we further split the short category into 2 subcategories (1-9 mins and 10-15 mins) so that bouts <10 minutes and >10 minutes can be compared (at the request of reviewers).”

Confounding by prevalent disease has not been adequately accounted for and the compositional nature of the time-bound data should be ideally complimented with analyses which account for that in a different way.

Please see our next response, and the response on page 8 of this document.

Specific comments 
Number of diseases at pre-baseline is a poor way to account for confounding by prevalent disease; you should use binary indicators of each disease as they have different impact on both activity and mortality. Moreover, as the accelerometer measurement was done about 5 years after the main study baseline, you need to use the routine health data, eg Hospital Episode Statistics which is available in UK Biobank, to reclassify disease status at the accelerometer baseline used in this analysis.

Thank you for this suggestion. We have now changed our covariates to include binary variables for three disease types: cardiovascular diseases, cancer and respiratory diseases. We use the date of first occurrence of each of these to identify those with the disease prior to accelerometer wear. This change had no material impact on our results.

We have updated the methods and results throughout, including changing this in the methods

(lines 279-281) from:

We used the number of baseline self-reported cancer and non-cancer illnesses as measures of baseline general ill-health”

To:

“We used three indicator variables for existing cardiovascular diseases, cancer and respiratory diseases prior to accelerometer wear as measures of baseline ill-health.”

It may be worth pointing out more clearly in the confounding section that you are using age at the time of accelerometer wear (and not at main study baseline).

Thank you, we were using age at assessment centre but agree age at accelerometer wear is more appropriate so have changed this. We have added the following emboldened text to make this clear (line 273):

“We considered the following to be likely confounding factors (based on their known or plausible effects on physical activity and mortality): sex, age **at the time of accelerometer wear**, ethnicity …”

Seasonal adjustment of accelerometer wear is better achieved using cosine functions as previous Biobank analyses have done.

Thank you for this suggestion. We have now updated our analysis to use cosine terms as covariates to account for seasonality (with no material impact on our results).

In the methods (lines 284-298) we have changed the text to:

“We therefore derived two variables denoting the day of the year on which the accelerometer was worn using the cosine function approach, defined as c1=cos(2*pi*d/365) and c2=sin(2*pi*d/365) where *t* is the day of the year accelerometer wear began [18]. We included c_1_ and c_2_ as covariates in our models, to reduce the variation in activity exposure variables.”

For ease of interpretation, we have retained the four seasons in the summary statistics (Table 1).

Did you calibrate the accelerometer readings to local gravity, eg using the approach described by van Hees? Raw data from this sensor is uncalibrated and calibration will greatly reduce noise and can be performed on almost all individual accelerometer files. Your flowchart suggests that you may have performed this but then again maybe not as you do not loose any data in this step.

Thank you for pointing this out. We used the UK Biobank accelerometer tool and field 90016 [https://biobank.ctsu.ox.ac.uk/crystal/field.cgi?id=90016] to identify those with poor calibration. There are only 11 participants with a calibration problem, and 2 of those were also identified as having a data problem so were removed in the previous step. This leaves 9 participants with a calibration problem, which due to a coding error were not removed from our sample. We have now rectified this issue, updated the participant flow diagram (Figure 1), and rerun our analyses without these participants (with no impact notable on our results).

Your hybrid approach of vector magnitude-based MVPA classification, combined with separation of the non-MVPA component into the other categories by the activity type classification method seems very appealing - do you have any evidence of its validity, though?

The classification of the sedentary and sleep categories using the machine learning model were validated during that model – ours only excludes minutes >=100mg from these categories as these are assigned to MVPA instead. Further work is needed to compare the two approaches of predicting activity categories but we are unable to do this since we do not have labelled data (i.e. accelerometer data with activities assigned for each timepoint). We have now added a comment about this in the discussion section of the paper (lines 714/715):

“Further work is needed to compare the types of mis-classifications of the hybrid and ML-only approaches.”

Note also that the activity classification uses a number of accelerometer features, some of which are orientation-specific; there are two main orientations of the accelerometer measurements in UK Biobank (as two versions of the monitor was used) and possibly an additional actual orientations owing to participants turning the puck inside the wristband upside down or reverse-lateral and wearing it on left or right wrist (all of which makes it challenging to reference accelerometer measurements to the anatomical coordinates). Suggest use version of activity classification without orientation-sensitive signal features.

We are unsure what the reviewer means by ‘two versions of the monitor was used’– please can they point us to documentations describing this? As far as we are aware, UK Biobank used the Axivity AX3 accelerometer for all participants.

We are using an existing machine learning model (Doherty, 2018) to predict activity categories, not training our own machine learning model to predict activity categories. Improving the machine learning predictions is not within the scope of this work.

We feel that turning the puck inside the wrist band upside down is unlikely, since it is already in position when participants receive it, and the puck has clear signs saying which way up it should go, should they remove it temporarily. Participants are instructed to wear it on the wrist of the hand they usually use to write, so it could be worn on either the left or right wrist. We have now added this possibility as a limitation in the discussion (lines 707/708):

“For example, the model uses some orientation specific movement variables and it is possible that some participants wore the accelerometer in an incorrect orientation.”

You collapsed the time-series to 1-min resolution of activity types before any further analysis? That is fine but you should justify this, particularly given your research question here! Note, epoch resolution and bout quantification interacts, see for example paper by Orme et al. Also note that working with 1-min data would mean that your intensity distribution estimates are different to those reported using the derived 5-sec movement intensity data available in the data showcase - again, this is fine but should be highlighted when comparing to other work.

Yes, bout summary variables depend on the definition of the bouts, and ours are based on a 1-minute epoch definition. We have now made this clear in the paper (lines 720-722):

“We used 1 minute epochs to derive activity bouts, but using a different epoch definition may affect the values of derived bout variables and hence our results [26].”

It is fine to use 10-min increments as the unit of time substitution in the analysis of association and your definitions of sedentary bout exposures are fine too. However, you should revisit the categorization of MVPA bout durations for defining these exposures; you must have one category boundary at 10-min (below and above), and it would make sense to have at least a few in the low range where all the variance is! Given you epoch decision and current guideline debate, I would suggest one category should be 1-min, then perhaps 2-9 min, then 10-15min and the remaining ones you already have (although I would probably collapse those if it was me). Your downstream analytical approach requires these to be mutually exclusive but it would actually be better to think about them as nested variables; at least 1-min duration, at least 5-min duration, at least 10-min duration, and so on - please reflect and respond.

Regarding the category boundaries of the MVPA bout strata, we have now updated these. Please see our response at the top of page 5 of this document.

We model our activity variables in terms of a coupling of activity categories, to reflect the way, when a person increases time in one activity category, they have to decrease one or more other activity categories by the same amount. If the reviewer feels a nested structure is more suitable, please can they justify this? We have also now conducted sensitivity analyses using an alternative approach (using isometric log-ratio transformed activity categories) and these results are consistent with our main analysis.

You have conducted a time compositional analysis of a bounded phenomenon (24 hours in a day), which makes assumptions about linearity in a constrained variance space; consider supplementing your time substitution analysis with a compositional data analysis or multivariate pattern analysis.

Thank you, we have now added an analysis using the isometric log ratio transformation.

In the methods on lines 415/416 we have added:

“We also conducted sensitivity analyses using the isometric-log ratio transformation, which accounts for the compositional nature of time spent in activity categories [25].”

In the results on lines 622/623:

“Results using isometric log-ratio transformed activity variables were consistent with our main analyses (S10-12 Figs).”

Mortality results have been updated in 2020 and you should therefore rerun your analysis for greater power.

We have updated our analysis to use the latest mortality data. Date of censoring is now 31^st^ December 2019. Our sample now includes 1,615 deaths, compared with 761 in the previous version.

Suggest include sensitivity analysis excluding those who died within the first year or two to account for potential reverse causality / confounding by ill-health (I think the 5 years you are mentioning in passing in the discussion is a bit extreme; this has been suggested by those who do not understand regression dilution).

We appreciate the importance of potential reverse causality / confounding by ill-health, and have now included a sensitivity analysis, starting follow-up one and two years after date of accelerometer wear (hence removing participants who died within this period from our analyses).

We now describe this sensitivity analysis in the methods on lines 413-415:

“We conducted sensitivity analyses for all models, starting follow up 1 year and 2 years after the start of accelerometer wear, to investigate whether our results may be biased by participants reducing their activity due to existing ill-health.”

In the results for overall time in activity categories (lines 543-544):

“Results attenuated towards the null when starting follow-up 1 year and 2 years after accelerometer wear (S5 Fig).”

And in the results for bout length (lines 567/568):

“Results starting follow-up 1 and 2 years after accelerometer wear were consistent with our main analysis (S8 Fig).”

In the discussion (lines 730-751):

“Residual confounding could also occur due to undiagnosed underlying chronic disease which could result in being less active and more sedentary, and be associated with increased mortality, particularly in the early years of follow-up. To explore this we conducted sensitivity analyses starting follow-up one and two years after accelerometer wear. Results from these analyses showed some attenuation towards the null for our overall time spent in activity categories, which may suggest our results are biased by confounding with existing ill-health, but might also be explained by any true effect of activity on mortality being short-lived. Longer follow-up time, further sensitivity analyses starting follow-up 5 years after accelerometer wear, and repeat assessments of physical activity, would help to ensure associations are not due to confounding via existing ill-health, and to explore the impact of changes in activity levels and whether any beneficial effect of activity might be short-term.”

Your findings do NOT directly support the abolition of the 10-min MVPA bout requirement as you have not compared associations for <10-min bout MVPA with those for 10-min or longer bouts!

Thank you, we have now amended our bout length categories to make them directly comparable, please see our response to your first comment about this at the top of page 5 of this document.

Previous work on time substitution in Biobank showed broadly similar mortality associations using the self-reported variables - maybe worth comparing your results to?

Thank you for this suggestion. We had already considered assessing self-reported variables and have not done this for the following reasons. First, our work using a compositional approach that is based on transferring time between one activity categories and another. For this we use the time spent in bouts of each duration category (1min, 2-9 min etc). UK Biobank self-reported data includes fields (IDs 981, 1001, and 3647) that describe bouts pertaining to bouts of ‘walking for pleasure’, ‘strenuous sports’, and ‘other exercises’, for example asking “Each time you did strenuous sports, about how long did you spend doing it?”. However, these are poor representations of time spent in bout durations as they are a summary of the average bout duration across all bouts – participants had to select one category rather than providing a distribution across bout lengths (which would more directly compare to the objective data we use in our analysis). For example, a person who does short, medium and long ‘walking for pleasure’ bout lengths would likely respond with a medium bout length, thus is not distinguishable from participants that only do medium bouts (not short or long bouts).

Second, there are two fields (IDs 884 and 904) where participants were asked “In a typical WEEK, on how many days did you do 10 minutes or more of moderate physical activities like carrying light loads, cycling at normal pace?” and “In a typical WEEK, how many days did you do 10 minutes or more of vigorous physical activity?”. These are meant to capture continuous bouts of 10 minutes or more, buts this is only clear from the help information e.g. “Count the number of days in a week that you do moderate physical activities for at least 10 minutes continuously at a time“. If a participant does not click on the help information to gain this information they would likely not realize this question is about activity bouts specifically.

Third, there are no self-reported variables describing length of sedentary bouts.

Our use of objective physical activity is a strength of this study, as we describe on lines 684/685:

“Strengths of this study include the large sample size and use of accelerometer data rather than self-report to measure activity, and the prospective nature of the study.”

**Reviewer #2:**

This is a nice study examining associations between physical activity and mortality. The study includes a large sample of UK adults (~80k), measured with accelerometry. This is among the largest studies that include accelerometry in a sample of adults. I do have a few concerns, perhaps the most relevant is the short time of follow-up.

Major:
1 - Participants were assessed in 2013-2015 and have follow-up through 2018. Follow-up time is expected to range from 3-5 years which can be a concern. It is not clear how limited follow-up might have impacted the results but it would be helpful to see sensitivity analyses for England/Wales vs Scotland, which seem to have different follow-up times and also after excluding first 2-3 years of follow-up.  It would also help if the authors include a sensitivity analysis excluding individuals with poor health or with major chronic conditions.

We have now updated our analyses to use the latest death data, more than doubling the number of deaths. Follow-up is now an average of 5.1 years [SD=0.73].

As end of follow-up is now the same (31^st^ December 2019) for those in both England and Wales, we are not able to perform the suggested sensitivity analysis for England/Wales vs Scotland.

We have now included an additional sensitivity analysis, excluding the first year and two years of follow-up, to assess whether our results may be biased by confounding with ill health.

We have now also improved our covariates, accounting specifically for cardiovascular diseases, cancer and respiratory diseases in our models – please see our response to reviewer 1 on page 5 of this document. We prefer this to excluding individuals of poor health as that would reduce our sample size, and mean our results are applicable to only healthy individuals.

2 - The study also includes a rather limited set of confounders. Important confounders such as diet and physical mobility limitations were not included. These are important confounders that can add beyond the ones that were included (eg, BMI). This issue of unmeasured confounding is problematic, particularly given the relatively weak associations that were found for MVPA-mortality.

We believe that diet is most likely to affect activity via BMI, and we have accounted for BMI in our analyses.

We have improved our analysis by adjusting for specific diseases (cardiovascular diseases, cancer and respiratory disease), which we believe to be the mostly likely health issues to confound associations between activity and mortality.

We agree that mobility limitations may confound the associations between activity and mortality. However, we are not aware of any appropriate variables in UK Biobank that reflect this, as the fields on disability (6146 and 2188) are general rather than identifying those with a mobility limitations specifically. We have now added this to the limitations section of the discussion on lines 726-728:

“For example, it is possible that mobility limitations, or little access to green space or facilities to be physically active, might be related to less time spent in light or MVPA and more sedentary behaviour, and also to increased risk of mortality during follow-up.”

Minor:
1 - Include follow-up information earlier in the paper (in the all-cause mortality section).

We have now moved the follow-up information to the all-cause mortality section of the paper (lines 267-269).

2 - Is there any proxy for mobility limitation that could be used as an additional confounder?

Thank you for this suggestion, we respond to this point above.

3 - About the MVPA bouts: I was expecting to see bouts of 1-10 min; 11+, etc. The intro (appropriately) address the discussion of 10+ bouts but the categorization used in the paper (15+) is not aligned with this bout criteria.

Also, after looking at the bout distributions, looks like there is not much data for longer bouts. The 1-15min bouts is a pretty long bout - it represents the majority of total MVPA (almost 90% by looking at table S2). This category includes non-bouted and bouted PA (to a max of 15min) given that the lowest resolution is 1-min epochs. Non-bouted and bouted activity should be separated here. It would seem more reasonable to use 1-4, 5-9, 10+, etc.  or 1-9, 10+. Also, were interruptions allowed when creating these bouts?

Thank you for this suggestion. We have now amended our MVPA bout categories by dividing our 1-15 minute bout category into three: 1 minute, 2-9 minutes, and 10-15 minutes. Please see further details in our response to reviewer 1 at the top of page 5 of this document.

Interruptions were not allowed, as stated on lines 336/338:

“For each participant we identified contiguous sequences of 1-minute epochs with a given activity category; these are referred to as activity bouts and can be of any length so long as the participant remains in the same activity category”.

The longer bouts (16min +) is likely skewed and makes me doubt there is enough data here to test these associations.  How many people reported PA in each of the bout levels? Showing these distributions and amount of '0s" will tell how much data there is for this particular analysis.

We include the median and interquartile range in Supplementary table 1. We have now also added new columns to this table, showing the number of non-zero values for each activity variable. While the 16-40 MVPA bout category is heavily zero-inflated, because our study uniquely has such a large sample (N=79,507) there are still 51,739 participants with non-zero values, making this analysis a worthwhile contribution to the literature.

Finally, still related to bouts - looks there is pretty much no data on 41+ bouts of PA. Pease show the distribution for this as well.

As described above, we give the median and interquartile range in Supplementary table 1 and have now also included the number of non zeros for each activity variable. While many participants do not have any MVPA bouts of length 41+, there are still 13,206 participants that do. Given the novelty of assessing this duration of bout length (and to keep consistency with our analysis of sedentary bouts which have much more data at this duration) we felt it was beneficial to include this bout length category – the size of our study makes assessing this longer bout length category feasible for the first time, and we feel this is a major strength of our study.

4 - Include an explanation for using age as time metric as oppose to time of follow-up. I wondered if truncating birth at July 1st results in losing the benefit of using age as time metric.

The UK Biobank has a fairly wide age range of participants, e.g. at recruitment participants were between 40 and 69 years old, but fairly short follow-up time. This makes it more appropriate to use age as the x-axis in our survival models, so that the hazard is modeled as a function of age rather than timepoint (i.e. participants of the same age are deemed more comparable than participants at the same point in follow-up time).

5- Please include a Table 1 with information on demographics and follow-up time. This in the suppl material but it would be helpful to see this in the main paper. Also, Table S1 shows there were 538 events which conflicts with ~700 reported in the main text. Please clarify.

We have now moved Table S1 to the main paper, now Table 1 on page 14.

The lower number of events in this table is because, in order to compare the number of deaths for participants without versus with accelerometer data, we use the last date of accelerometer wear time (across all participants) as the start of follow-up for all participants. This is to enable fair comparison across these groups given that participants without accelerometer data do not have an accelerometer wear time date. We included a footnote (d) to explain this. We have also now made this clearer by including an extra row, containing the number of deaths of our analysis with NA in the cells comparing across samples.

6 - In the discussion section, references in support of excluding the bout criteria (as per old guidelines recommendation) can't be supported here as bouts were not generated to test this assumption. See earlier comment and refine bout classifications.  

We have now amended our bout categories as described at the top of page 5 of this document. This means that we are now directly testing the question of whether 10min+ bouts of MVPA are more beneficial compared with shorter bouts.

7 - The following statement shows up a couple times in the paper but its not accurate:

"There are no previous studies assessing the association of MVPA bout length with mortality"

Here is a study that should be cited/discussed for this purpose:

Moderate‐to‐Vigorous Physical Activity and All‐Cause Mortality: Do Bouts Matter?
Saint-Maurice PF et al. JAHA 2018

Thank you for pointing us to this paper. We have now amended the text throughout. In the introduction we have now amended this on lines 176-178:

“Of three studies in adults, two found no notable association of MVPA bout length with their respective outcomes; cardiovascular risk factors (N=2,190) [7] **and all-cause mortality (N=4840) [8]**.”

And on lines 202-204:

“To our knowledge, our study is the largest observational study of activity bouts to date and **the second** to assess associations of MVPA bout length with all-cause mortality.“

In the discussion (lines 675-677):

“To our knowledge, there is only one existing study that assessed the association of MVPA bout length with mortality; it was considerably smaller than our study (N=4,840) and consistent with our findings, found no strong evidence of association between MVPA bout length and mortality [8].”

8 - I mentioned this before but suggest that the discussion on short follow-up be expanded as a critical limitation. I also suggest additional sensitivity analyses. Eg, excluding people with chronic conditions.

The following paper might be helpful for this purpose:

<https://pubmed.ncbi.nlm.nih.gov/32472927/> 

We have now expanded our discussion of the follow-up and possibility of confounding via ill-health, changing the text on lines 730-751 from:

“Sensitivity analyses restricting follow-up to those alive 5 years after accelerometer wear would help to ensure associations are not due to confounding via existing ill-health, but follow-up periods in UK Biobank are currently too short to be able to do this.”

To:

“Residual confounding could also occur due to undiagnosed underlying chronic disease which could result in being less active and more sedentary, and be associated with increased mortality, particularly in the early years of follow-up. To explore this we conducted sensitivity analyses starting follow-up one and two years after accelerometer wear. Results from these analyses showed some attenuation towards the null for our overall time spent in activity categories, which may suggest our results are biased by confounding with existing ill-health, but might also be explained by any true effect of activity on mortality being short-lived. Longer follow-up time, further sensitivity analyses starting follow-up 5 years after accelerometer wear, and repeat assessments of physical activity, would help to ensure associations are not due to confounding via existing ill-health, and to explore the impact of changes in activity levels and whether any beneficial effect of activity might be short-term.

”

We feel that adjusting for chronic health conditions in our models by including these factors as covariates is a more appropriate way of accounting for these, rather than excluding these people from our analysis, as that would mean our sample is less representative of the UK population and reduces statistical power. We have now also included sensitivity analyses starting follow-up 1 and 2 years after accelerometer wear to further explore confounding by existing ill-health.

We have updated follow-up so it is now substantially longer than before, with the date of censoring not 31^st^ December 2019 rather than 30^th^ November 2016 (Scotland) and 31^st^ January 2018 (England and Wales). Our sample now includes 1,615 deaths, compared with 761 in the previous version.

Given the weak associations with MVPA, a small degree of confounding is likely to attenuate these effects to become null. This should be part of the discussion here as well.

We discuss residual confounding in the discussion on lines 724-728. We have now extended this adding the following emboldened sentence to this paragraph:

“While we accounted for known, measured confounders, our analyses may be biased by residual confounding. **It is possible that adjustment for other confounders might attenuate results (e.g. of overall time spent in MVPA) to the null.** For example, it is possible that having mobility limitations, or little access to green space or facilities to be physically active, **might be related to less time spent in light or MVPA and more sedentary behaviour, and also to increased risk of mortality during follow-up.**”

**Reviewer #3:**

I confine my remarks to statistical aspects of this paper. These were very well done, but I have a few questions and comments before I can recommend publication.

Thank you for your positive feedback on our statistical analysis.

First, I wish more of the technical details were in the main paper, but I suppose that is a matter for the editors; it isn't a mistake to put it in a supplement.

As described below, the machine learning model was published previously, and the evaluation performance we refer to was conducted as part of that study (Doherty, 2018) and is not part of our study. We have now made this clearer in the paper and added some more detail as described below.

Line 160-161  I am curious about how sleep was distinguished from sedentary activity. Even if the technical details are given in an appendix, I think a plain language summary of this would be useful; after all, people do move in their sleep and some people lie still (but awake) for some time before sleeping.  In fact, a plain language summary of all the levels would be useful.

We use a previously published machine learning model to predict the activity categories. The machine learning model was able to distinguish between sleep and sedentary very well. Their paper states that 91% of sleep minutes were correctly predicted as sleep, with 8% incorrectly predicted as sedentary (and 1% as another category). Also, 81% of sedentary minutes were correctly predicted as sedentary, with 6% incorrectly predicted to be sleep (and 14% as another category).

We describe this machine learning approach on lines 318-332, and have added the following emboldened text to add further detail:

“We assigned each 1 minute epoch (interval) of accelerometer data to an activity category – either sleep, sedentary, light or MVPA. To do this we made use of a previously published machine learning model [18] that predicts activity categories (sleep, sedentary, walking, light and MVPA) from accelerometer data, that was trained using accelerometer data captured in free-living conditions and labelled with ‘ground truth’ activities from accompanying videos and the Compendium of Physical Activities **determined using a body worn camera** [19]. **The machine learning model predicted the activity categories with varying levels of success. For example, while 91% of minutes spent sleeping were correctly classified as sleep, only 25% of light activity minutes were correctly classified as light activity**.”

Line 165-168 
    1.  What was the "gold standard"?  That is, you say some periods were misclassified. But how was the right classification determined? And, if the right category could be determined, why wasn't it used? It seems to me there are going to be some murky periods no matter what you do. (e.g. time spent just before sleep; time spent going to the bathroom in the  middle of the night; activity that is on the border between moderate and light; and so on.

The ground truth activity, used to train the machine learning model, was captured using a body-worn camera, and labelled by the researcher team of reference [20] (Doherty et al, 2018).

The misclassification we are referring to is that in the evaluation conducted in the previous study not conducted by us. We have now made this clearer in our paper, changing the text on lines 342-346 from:

“There are two reasons we used the hybrid approach as our main analysis: 1) the degree of misclassification of the machine learning model for MVPA estimated in the study publishing this model [18] (e.g. only 58% of MVPA minutes were predicted correctly as MVPA), and 2) the activity categorization in [18] included a separate walking category whereas we consider brisk walking to be moderate activity and slow walking to be light activity, with no separate walking category.”

To:

“There are two reasons we used the hybrid approach as our main analysis: 1) the degree of misclassification of the machine learning model for MVPA **estimated in the study publishing this model** [20] (e.g. only 58% of MVPA minutes were predicted correctly as MVPA), and 2) **the activity categorisation in [20] included** a separate walking category **whereas we sought to categorise** brisk walking as moderate activity and slow walking **as** light activity**, with no separate walking category**.”

   2.  Given that you state some of the misclassification (58% of MPVA) I'd suggest giving all the results, for both models.

The evaluation of the machine learning models was conducted by the authors of the machine learning model (as described above) (Doherty; 2018). We therefore include a summary of this performance and refer the reader to their paper, as described above.

Also, as a statistician, I'm just curious why three different programs were used --- it's fine to do so and all the programs are good ones, I'm just wondering

Most of the project was conducted in R. I did the confounder analyses in Stata, just because I find Stata convenient to explore variable summaries. I plot my results in Matlab as I personally find it easy to make them look nice, and also easy to customize using Matlab. The UK Biobank accelerometer tool is third party software and written in Java.

We have made all our code available in a GitHub repository to aid reproducibility: <https://github.com/MRCIEU/UKBActivityBoutLength/>.

Overall a very interesting analysis!

Peter Flom
