# Supplementary material for: Association of physical activity intensity and bout length with mortality: An observational study of 79,503 UK Biobank participants
Source: PLoS Med. 2021 Sep 15;18(9):e1003757. doi: 10.1371/journal.pmed.1003757 (PMC8480840; doi:10.1371/journal.pmed.1003757)
Supplement: Attachment — Submitted filename: reviewer-responses-20201218.docx [file pmed.1003757.s023.docx]

We thank reviewers 2 and 3 for recommending acceptance of our paper, and reviewer 1 for their helpful comments.

Requests from the editors:

*We'd recommend that the data availability statement provided in the article submission system is updated to include the github links for code availability (provided in the authors' response and main text).

The data availability statement has been updated to include the GitHub link for our code repository.

Reviewer #1:

The revisions have improved this paper substantially, particularly the update of follow-up and the re-specification of bouts around the currently discussed bout duration boundary of 10 minutes. There are, however, a few outstanding concerns remaining as outlined below.

Title: You have not assessed change in physical activity but between-individual differences - change title accordingly (eg, delete "changing").

Thank you, we have now removed “changing” from the title. It now reads “Association of physical activity intensity and bout length with mortality: a prospective study of 79,503 participants in UK Biobank”.

Abstract:
It is not a trial so your conclusion should be that activity is associated with lower mortality, not "reduced" mortality. Same issue with "increased" mortality risk - it should read "higher". Make global check for causal language.

Thank you. We have amended these in the abstract and reviewed the manuscript to remove causal language. For example, in the author summary on page 4:

“We found that more time spent in MVPA **was associated with lower** mortality risk irrespective of whether it replaced light activity, sedentary or sleep time; more time spent sedentary **was associated with higher** mortality risk, particularly if it replaced MVPA.”

In the discussion on page 21:

“Our study confirms a strong association between active time and **lower** mortality, **particularly for** MVPA compared with light activity.”

Introduction:
There are other papers from the cohort reporting on the general association between accelerometry-measured exposure and mortality. For example, a recent paper by Strain et al (Nature Medicine, 2020) reported on the associations between accelerometry-measured activity and mortality using the same dataset but did not look at the potential importance of bouts - suggest reword intro slightly to leap from there.

We have now added the following text with citations on lines 163-167:

“To date, several studies have assessed the relationship between MVPA and all-cause mortality in UK Biobank [17–19]. These studies did not assess associations with bout length. The aim of our study is to examine whether mortality differs depending on time spent in different activity categories (e.g. including sleep and sedentary, not just being physically active), and whether time spent sedentary or in MVPA is accumulated in longer versus shorter bouts.”

Note that the recently launched WHO guidelines for physical activity have also lost the 10-min bout requirement, and there is increased recognition of light activity too - may allow you a slightly elevated pitch to refer to global guidelines rather than just US/UK national?

Thank you. We have now updated the introduction to include the WHO guidelines, on page 5:

“Policy in the UK recommends that people accumulate 150 minutes each week in moderate physical activity or 75 minutes in vigorous activity **[3], while policy in the US and World Health Organization (WHO) guidelines have been recently updated to recommend ranges (150-300 minutes each week for moderate intensity and 75-150 minutes for vigorous intensity) rather than minimum amounts alone [4] [5]**. Until recently, the advice also stated that activity should be accumulated in bouts of 10 minutes or more, but **this has now been removed from UK, US and WHO guidelines [3] [4] [5]. These changes were based on evidence from cross-sectional, prospective cohort and randomized trials. For example, the removal of minimum bout length from WHO guidelines was based on a systematic review [6] of 27 research studies: 13 cross-sectional studies, 2 prospective cohort studies, 1 non-randomized trial and 11 randomized trials. The trials had small sample sizes (all** $\boldsymbol{\leq}$ **255) and short term follow-up (**$\boldsymbol{\leq}$ **18 months). The largest sample size among the included prospective cohort and cross-sectional studies was 6,321.**”

Methods:
Invitation to join the accelerometry subsample was not random; everyone with a valid email in the cohort were invited, except those currently taking part in substudies.

Apologies, we have now corrected this sentence on lines 197-200, from:

“In 2013 a subsample of 240,000 UK Biobank participants were randomly selected from those who had provided valid email addresses, and invited to participate in the accelerometer sub-study. Participants of one assessment centre (3,797 participants; 0.76% of the cohort) were excluded from being invited as they had been invited to participate in pilot studies [20].”

To:

“In 2013, participants who had provided valid email addresses were invited to participate in the accelerometer sub-study, apart from the participants of one assessment centre (3,797 participants; 0.76% of the cohort) who were excluded due to participant burden concerns as they had been invited to participate in pilot studies [20].”

Thanks for confirming that you have calibrated your accelerometer data to gravity; they would otherwise not have been valid and we could stop right there! Please include appropriate citation for the method - the Biobank tool uses the van Hees method (with temperature correction) and other tools use either that or the Lukowic method, both of which are sufficient but you do have to use one.

We have now included a separate section describing the accelerometer data preprocessing using the UK Biobank tool, that contains this detail on page 9:

“Accelerometer data pre-processing

We used the UK Biobank accelerometer analysis tool (available at <https://github.com/activityMonitoring/biobankAccelerometerAnalysis/>) [20,22,23] to pre-process the accelerometer data and derive summary activity variables for each 1-minute epoch in each participants accelerometer time-series. The steps conducted by this tool include resampling x/y/z axes to 100Hz, calibration to local gravity [24], noise and gravity removal, epoch generation – including both average vector magnitude and machine learning predictions of physical activity categories for each epoch – and non-wear detection. The machine learning model [22] predicts activity categories (sleep, sedentary, walking, light and MVPA) from accelerometer data. It was trained using accelerometer data captured in free-living conditions and labelled with ‘ground truth’ activities from accompanying videos and the Compendium of Physical Activities determined using a body worn camera [25].“

To clarify the point about accelerometer axis orientation: All accelerometers used were Axivity AX3 but there are two versions of that. The first batch of accelerometers used in UKB had the X and the Y axes swopped around in the casing, compared to subsequent batches used. There are no other differences as the chip is the same; it simply means a rotation of 90 degrees on the wrist mount. The meta data of the .cwa files includes this information through its serial numbers but you can also see it in the data as the axis angles (pitch and roll) with gravity (low-frequency component in the data) differ between the two batches. The activity classification work by Willett et al (Sci Rep 2018 - the correct citation for the method used in Doherty et al, 2018) used the orientation of the X and Y axes of the more recent batch, so is directly applicable to that part of the UKB data but not directly to the data collected with the first batch of monitors. Although only some of the several signal features used in the activity classification method are orientation-sensitive, the optimal application of the method requires remapping X acceleration to Y and vice versa as an initial step for those who were measured with the earliest version of the accelerometer.

Please see response at the bottom of page 5 of this review document, where we provide the updated text regarding possible orientation differences.

We would be interested to hear if these details on the orientation differences between batches are documented anywhere (several authors looked through all of the UK Biobank documentation and contacting others who have used these data, and we could not find any mention of this in UK Biobank documentation and key papers).

The splitting of the MVPA exposure category around the 10-min boundary is excellent and will make the results of this analysis far more relevant to the current debate on the issue. I have to say, the other categories do not seem entirely logical to me, eg why split MVPA at 40 minutes and not 30 minutes? And why not split the sub-10 category further, eg at 5-min, as that is where all the behavioural variance is (and one notes that exact example of 5-min bouts in your summary statement)? And why insist on alignment of sedentary and MVPA bout durations when the notion of short and long are materially different for such behaviours?

Thank you for appreciating our efforts to implement the 10-minute boundary, as you suggested in your previous review. The reason we chose 40 minutes not 30 minutes was because often a person will do 30 mins of exercise and we wanted a boundary that would effectively group the ~30 min bouts into one, and the bouts definitely longer than 30 into another. We chose to use the same bout length strata for MVPA and sedentary bouts for consistency, although we do appreciate another approach would have been to use strata chosen specifically for MVPA and sedentary behaviour.

We have now added the following sentence to the discussion on lines 693-696:

“We chose to use the same bout length strata for MVPA and sedentary behaviour for consistency, but we may have had more statistical power by defining strata according to the distribution of bout lengths for each category (e.g. participants spent more time in longer (versus shorter) sedentary bouts and more time in shorter (versus longer) MVPA bouts).”

I don`t think you need to keep saying "at the request of the Reviewers" in the Methods as you have already stated it when describing deviations from your analysis plan but I refer to the Editor here for specific journal policy.

We prefer to make this clear in the paper, and the editor has said that it is fine to do this.

With respect to the approach of defining exposure as nested vs mutually exclusive, I appreciate the opportunity to do the reflections for you since you ask; it is certainly not wrong to conduct a mutually exclusive categorization analysis as you have done but given that the recent debate was not about which bout duration activity should have (short or long) to confer health benefits but rather if it should have a minimum duration of 10min or not, the analysis that more directly addresses that issue is one that contrast associations of exposures with and one without a minimum bout duration definition (and not a maximum one).

We began this work prior to the debate on the minimum duration – our original aim was to determine whether there was evidence of differential associations across bout length strata, i.e. whether one appeared more beneficial than another and for this we decided it was appropriate to use mutually exclusive categories. Thank you for confirming that you agree that our approach using mutually exclusive categories is not wrong.

Results:
Main table 1 doubles up as a drop-out analysis - fine although I would have preferred a clean main table of the data in the second column and then this one in supplement.

We have now moved the drop-out analysis to the Supplement (S1 Table). Table 1 (page 14) now only summarizes characteristics of participants in our sample, referred to on lines 366/367:

“Descriptive statistics of our main (complete days) sample are provided in Table 1.”

I see no more main tables?  Are supplementary table S3a and S4 not the most essential set of results? Or do figure 2 and 3 serve this function as main results? We are not helped by the formatting issue here - there is a dead-end bookmark in my review copy.

Yes Figures 2 and 3 display the main results of our paper, and this is the same information as the results in S4a Table and S5a Table. We have now added the following text to make this clear, in Figure 2 footnote on line 575:

“Results shown are also provided in S4a Table.”

And Figure 3 footnote on line 599:

“Results shown are also provided in S5a Table and S6a Table.”

We have now updated the Figure links so they are no longer showing an error on the submitted PDF, please see our response to the last comment on page 6 of this document.

Discussion:
Suggest delete "uniquely" from opening statement - it reads very odd and probably isn`t even true unless you caveat it somehow but that would make it worse.

We have removed ‘uniquely’ from this sentence (line 618).

Suggest make reference to global public health recommendations by the WHO instead - seems more convincing. It is not true that the removal of 10-min MVPA bout duration requirements from US and UK guidelines were purely based on trial evidence - observational epidemiological evidence such as the type you report here was also considered, including some of the papers you cite here yourself - please rewrite.

We have now updated the discussion to refer to WHO guidelines, on lines 622-626, from:

“These findings emphasize the specific importance of MVPA. They also support recent policy changes in some countries that have removed the suggestion that MVPA should be accumulated in bouts of at least 10 minutes [3–5]. Those policy changes were made on the basis of a systematic review of randomized controlled trial evidence but the trials were small, only looked at associations with continuously measured risk factors, and were all deemed to have high risk of bias in that review [7].”

To:

“These findings emphasize the specific importance of MVPA. They also support recent **changes to policy** **in UK and US and WHO guidelines** that have removed the suggestion that MVPA should be accumulated in bouts of at least 10 minutes [3–5]. Those policy changes were made on the basis of **cross-sectional, prospective cohort and randomized controlled trial evidence, but those studies were small (e.g. in the systematic review on which this change in WHO guidelines was based the largest observational study had 6,321 participants and the largest trial had 255 participants [6]).”**

Your points about the limitations of self-report are well made - in fact so well made that you should include them in your paper. The fact remains though that the overall conclusions about time in different intensities do align well with previous reports from the full 500k participants in the cohort with longer follow-up.

We have now included our points about self-reported data in the discussion on lines 666-671:

“This was possible because of our use of accelerometer data, and would not be possible using UK Biobank self-reported activity data. The UK Biobank self-reported data (and most other self-reported data) on activity bouts cannot be analysed in a compositional way because they do not include time spent in bouts of different length of each activity category (only average time spent in bouts for some activities, or the number of days the participant did at least 10 minutes of moderate or vigorous activity).”

With respect to the proposed text edits on the accelerometer orientation issue, it is wrong to blame this on the participants as those in the early batch most likely did what they were told to do with that version of the monitor (so they did not wear it incorrectly) - please rephrase, including making the point that your hybrid approach of using vector-magnitude-based MVPA mitigates against this being a major issue for most of your main analysis (as vector magnitude is not orientation-specific).

We were not meaning to ‘blame’ the participants, and have now reworded this sentence, and added an additional comment pointing out the orientation independence of AVM as an advantage for our hybrid approach, on lines 679-684:

“For example, the model uses some orientation specific movement variables and it is possible that the **accelerometer orientation varied between participants**. However, our main analysis used a hybrid approach where MVPA was identified using a threshold (>100 m-grav), since prediction accuracy for MVPA from the machine learning model was particularly low. **This also has the benefit that average activity (denoted using the average vector magnitude) used to define MVPA in our hybrid approach is orientation independent**.”

"… any true effect of activity on mortality being short-lived" - I can`t quite decide whether this is an unfortunate phrasing of words or rather smart or funny but if this was unintentional, you should probably rephrase it!

We have updated this to “any true effect of activity on mortality being short-**term**” on line 714.

An issue which requires further discussion is the fact that 1-min epoch data are 1-min bouts - maybe just say exactly that where you mention that it might change if you had used another epoch setting which is a bit vague.

We have now added the following emboldened text to this sentence on lines 696-700:

“We used 1 minute epochs to derive activity bouts **(e.g. a 10 minute bout is a set of 10 adjacent 1-minute bouts)**, but using a different epoch definition may affect the values of derived bout variables and hence our results [30].”

Also, whilst I agree that some previous analyses have not accounted for overall time in sedentary or MVPA, you have not accounted for intrinsic differences in the intensity of MVPA between bouts; it is perfectly possible that those who go for longer MVPA bouts are also those who run faster when doing MVPA! In the recent Strain paper from the cohort, total volume was a main driver of the association with mortality, with additional benefit suggested from increased fraction of that volume coming from MVPA. Your analysis does not account for volume differences and intensity differences within exposure categories (which also makes your cutpoint decision far more influential) but the previous analysis does - a paragraph to discuss this issue should suffice.

We agree that there could be differences in intensity of the MVPA bouts across different bout length strata, and also differences in physical activity volume (the product of intensity and time). We have now added the following discussion of this (citing the Strain paper) on lines 720-729:

“Our use of time spent in each activity category and activity bout length strata does not account for variability of activity levels within each of these. For example, participants spending more time in short MVPA bouts may have higher activity intensity levels within these compared to those spending more time in longer MVPA bouts). It also does not account for energy expenditure which may also differ within and across activity categories and bout length strata. Other recent work assessing the association of physical activity estimated energy expenditure (PAEE) with mortality, also in UK Biobank, found that higher overall PAEE was associated with lower mortality and that associations were stronger with an increasing time spent in MVPA [17].”

General formatting errors throughout for references to within-document items, eg figs/tables.

Apologies, these are now corrected in the paper, on lines 203, 365, and 551.
