# Supplementary material for: Association of physical activity intensity and bout length with mortality: An observational study of 79,503 UK Biobank participants
Source: PLoS Med. 2021 Sep 15;18(9):e1003757. doi: 10.1371/journal.pmed.1003757 (PMC8480840; doi:10.1371/journal.pmed.1003757)
Supplement: Attachment — Submitted filename: reviewer-responses-20210721.docx [file pmed.1003757.s024.docx]

We thank the editor for their comments and respond to each point below.

Requests from Editors:

Please adapt the data statement to: "... available from UK Biobank for researchers who meet the criteria for access to confidential data ..." or similar.

We have added the following emboldened text to the data statement:

“The data underlying the results presented in the study are available from UK Biobank [www.ukbiobank.ac.uk] **for researchers who meet the criteria for access to these confidential data**. Analysis code is available at [https://github.com/MRCIEU/UKBActivityBoutLength/].”

Please remove the word "prospective" from the title (we think that your study is a retrospective analysis of prospectively gathered data). So as to comply with journal style we suggest: "Physical activity intensity and bout length and mortality in 79,503 UK Biobank participants: a population-based cohort study".

We have now amended this in the manuscript and the online web form to:

“Association of physical activity intensity and bout length with mortality: an observational study of 79,503 UK Biobank participants”

At line 22, should that be "... less light activity ..."?

We have updated this to “less light activity”.

At line 24 and elsewhere, we suggest making that "sedentary time".

We have updated this to “sedentary time” on lines 24, 60, 271, 332, 334 and 335.

In the abstract, we suggest quoting the 95% CI and p values immediately after the point estimates, e.g., "... (HR 1.02 [95% CI 1.01 to 1.02, p<0.001] per 10 minutes more sedentary time ...)".

We have updated this in the abstract on page 2.

At line 28, we suggest removing "UK Biobank had a 5% recruitment response", as this can be mentioned in the main text.

We have removed the text “UK Biobank had a 5% recruitment response” from the abstract on page 3.

At line 32 please make that "was associated" as you are reporting findings, with the surrounding wording adapted to be consistent with this.

We have changed “is associated” to “was associated” on page 3.

At line 82, for example, please adapt the reference call-out to the style: "... amounts alone [4,5]." (noting the absence of spaces within the square bracket).

We have updated this on page 5.

Please restructure the paragraph beginning at line 109, where the elements of discussion should be removed or transferred to the Discussion section. We anticipate 1-2 sentences stating the aim and, to an extent, the methodology of your study. The sentence beginning at line 118 should be removed.

We have amended this paragraph on page 6 as requested:

“The aim of our study, also in UK Biobank, is to examine whether mortality differs depending on time spent in different activity categories (e.g. including sleep and sedentary, not just being physically active), and whether time spent sedentary or in MVPA is accumulated in longer versus shorter bouts. We use a novel analytical approach that addresses limitations of previous studies, to assess associations of overall time spent in different activity categories and bout length categories in terms of coupling more time spent in one category with less time in another category.”

You mention an analysis plan at line 124. Please include this as an attachment if available, referred to in the text.

The analysis plan was discussed via email and so is not available to include as an attachment.

At line 164 and elsewhere as appropriate, please use the style "follow-up".

Thank you. We have updated this throughout the manuscript.

We ask you to adopt more circumspect language in places. For example, at line 385 to avoid over-generalizing we suggest amending the wording to: "In this study, we found that time spent in MVPA was associated with lower mortality ..." or similar (with the tenses adjusted to match in the rest of the paragraph).

We have now amended the language in this paragraph on page 17 to be more circumspect.

Please use the journal name abbreviation "PLoS ONE" in the reference list.

We have amended this on page 23.

Please add "[preprint]" to any preprints cited, reference 29 perhaps being one.

This paper (now reference 26) is now published so we have updated this reference to:

“Groenwold RHH, Palmer TM, Tilling K. To adjust or not to adjust? When a “confounder” is only measured after exposure. Epidemiology. 2021;32: 194–201.”

Please remove the information on funding and the UK Biobank application from the end of the text. The former will appear in the article metadata in the event of publication, via entries in the submission form, and the latter can be moved to the Methods section.

We have removed the funding information on page 24 and moved the application number to the methods section (page 7).
