# Supplementary material for: Association of physical activity intensity and bout length with mortality: An observational study of 79,503 UK Biobank participants
Source: PLoS Med. 2021 Sep 15;18(9):e1003757. doi: 10.1371/journal.pmed.1003757 (PMC8480840; doi:10.1371/journal.pmed.1003757)
Supplement: Attachment — Submitted filename: reviewer-responses-20210803.docx [file pmed.1003757.s025.docx]

We thank the editor for their comments and respond to each point below.

Requests from Editors:

Does "income (pounds)" need to be specified in table 1?

Thank you, we have added “(pounds)” to this table. We have also added kg/m^2^ as the units for BMI.

Comments from Academic editor:

I note an issue with supplementary figures S10-12 which show the compositional analysis results (judged by some to be the correct way to examine the main question posed in this paper, so fairly essential). Specifically, the authors need to explain what the two curves in each colour or panel represents, and secondly comment on what the results actually show; the all-too-brief statement in the manuscript says that that these analyses show the same as the main results but in some cases they appear to show the exact opposite or indeed very complex U-shaped associations! This needs a proper description and a proper discussion.

Thank you for pointing out this issue. There was a technical issue with the figure plots which meant that on some devices the confidence intervals around the plot lines were not showing. We have now fixed this issue (by printing these figures to a PDF document), and you should now be able to see the complete figures. The confidence intervals are key to interpreting these plots – the ‘U’ plots the editor is referring to are due to a lack of statistical power, where the confidence intervals of these estimates are very wide and also consistent with linear associations.

We have added further explanation of the interpretation of these results to the footnotes of each of these figures. For example, the following text is provided in the footnote of figure 10:

“Each curve shows the hazard ratio (and corresponding 95% confidence interval) for removing the number of minutes specified on the x-axis, from the baseline category (specified in legend), and adding this amount of time to the comparison category (specified in the title of each plot). For example, the green line of the ‘sleep’ plot shows, for the positive values on the x-axis, the hazard ratio for less time in MVPA, coupled with spending more time sleeping. The linear relationship shown in this plot suggests that the association for doing e.g. 10 minutes less MVPA coupled with 10 minutes more sleeping is approximately half the association for doing 20 minutes less MVPA coupled with 20 minutes more sleeping.”

A minor point is to sharpen up and strengthen the statement in the summary of “what do these findings mean”: The authors have analysed the accelerometer data in 1-min time resolution, and so the shortest bout of any activity category is 1 minute. Although it is not directly reported anywhere (but perhaps should be), we can deduce that the most common “short” MVPA bout is very close to 1 minute, given that the median time spent in total MVPA is 92 min per day and the median time spent in short MVPA bouts is 72 min per day. Hence, the example of 5-min bout duration for MVPA is a bit odd and probably wrong or at least sending the wrong message; what we have learned from this analysis is that MVPA bouts as short as 1 minute in duration have benefits for human health – a more accurate and more powerful message, providing very strong support for the most recent health recommendations.

Thank you for pointing this out. We have now included this information in the Discussion section on page 19:

“Participants tended to spend relatively little time in MVPA **overall and have MVPA bouts of short duration (the most common bout length was 1 minute, which was the shortest possible bout length in our data)** so these estimates were imprecise.”

We have changed the ‘What do these findings mean?’ section to give 1-minute as the example bout duration on page 4.
